# Supplementary material for: Risk caused by the propagation of earthquake losses through the economy
Source: Nat Commun. 2022 May 25;13:2908. doi: 10.1038/s41467-022-30504-3 (PMC9132971; doi:10.1038/s41467-022-30504-3)
Supplement: Supplementary file 1 — Supplementary information [file 41467_2022_30504_MOESM1_ESM.pdf]

Supplementary information

Risk caused by the propagation of earthquake losses through the economy

J. A. León, M. Ordaz, E. Haddad, I.F. Araújo

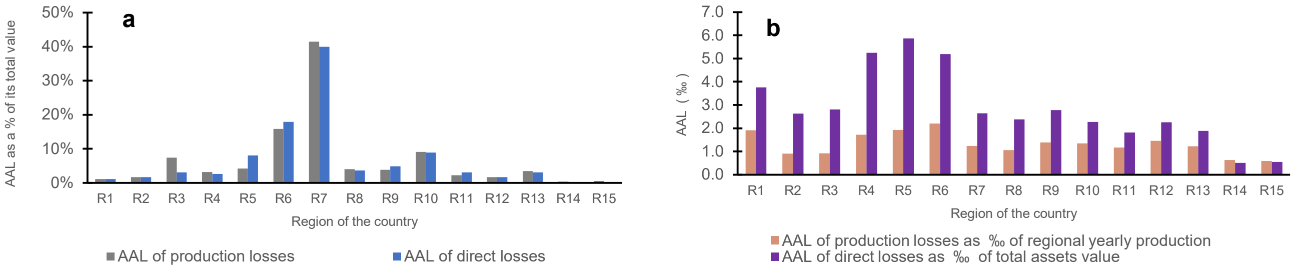

Supplementary Fig. 1 Average annual loss in Chile. Panel **a** shows the contribution of each region of Chile to the total AAL of both direct and production losses, respectively. Panel **b** exposes 1) the AAL of direct losses by region of the country as a fraction (per thousand) of its corresponding regional value of non-residential buildings 2) AAL of production losses by region as a fraction (per thousand) of its corresponding regional yearly production. AAL is presented as a percentage of its corresponding total value to see the influence of each region in the total AAL and as a fraction (per thousand) of its corresponding exposed value to see how risky each region is.

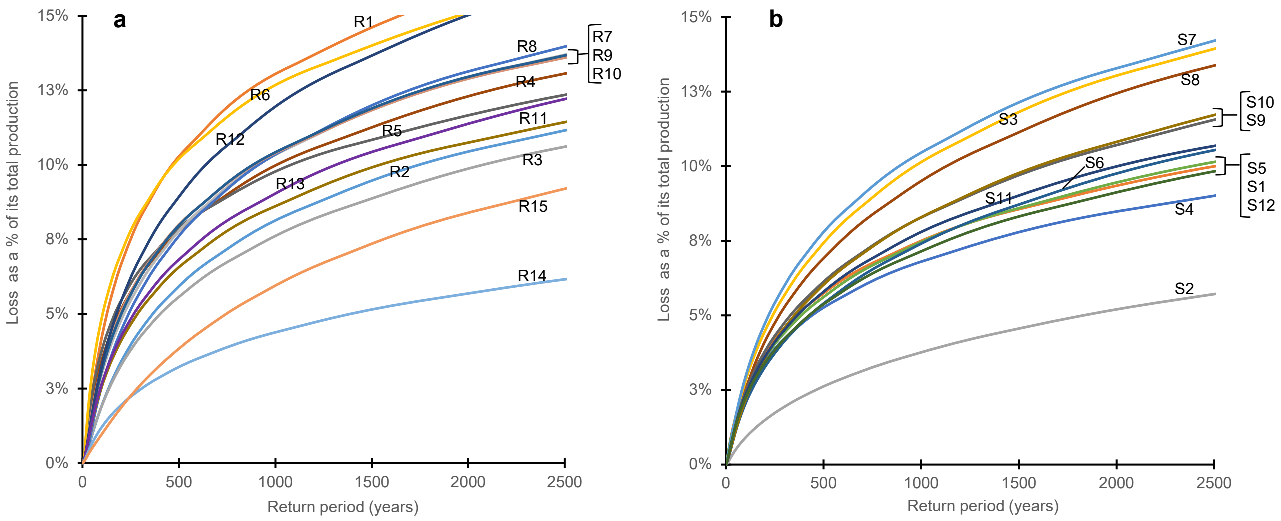

Supplementary Fig. 2 Production loss exceedance curves of Chile. Panel **a** shows the losses by region of Chile as a percentage of the corresponding regional yearly production, and panel **b** shows the losses by economic sector as a percentage of its corresponding sectorial yearly production.

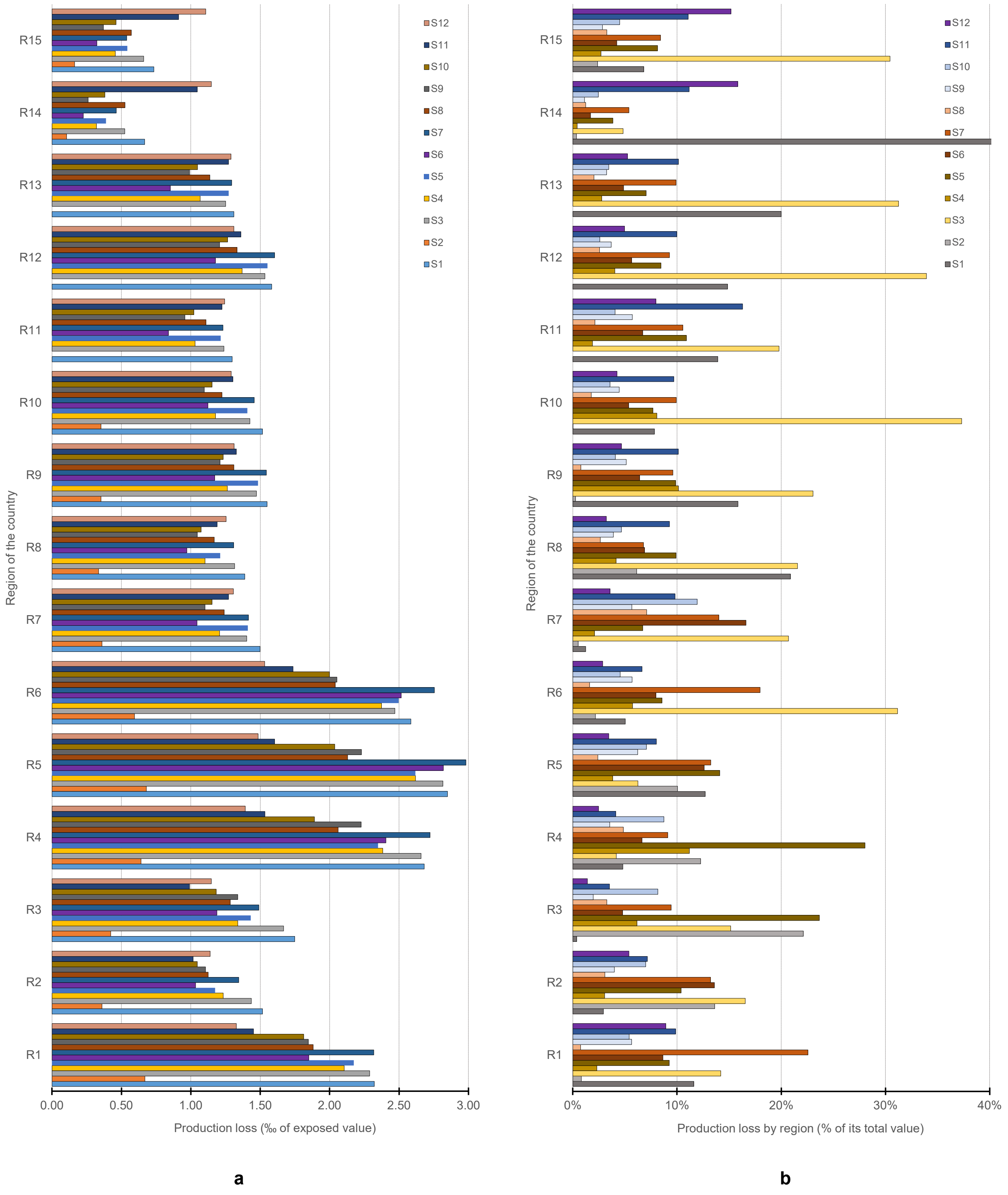

Supplementary Fig. 3 Average annual loss of production in Chile by economic sector at the regional level. Panel **a** shows the AAL as a fraction (per thousand) of its sectorial/regional yearly production to see how risky each sector is within the corresponding region. Panel **b** shows the contribution of each economic sector to the corresponding regional AAL of production for the 15 regions of Chile.

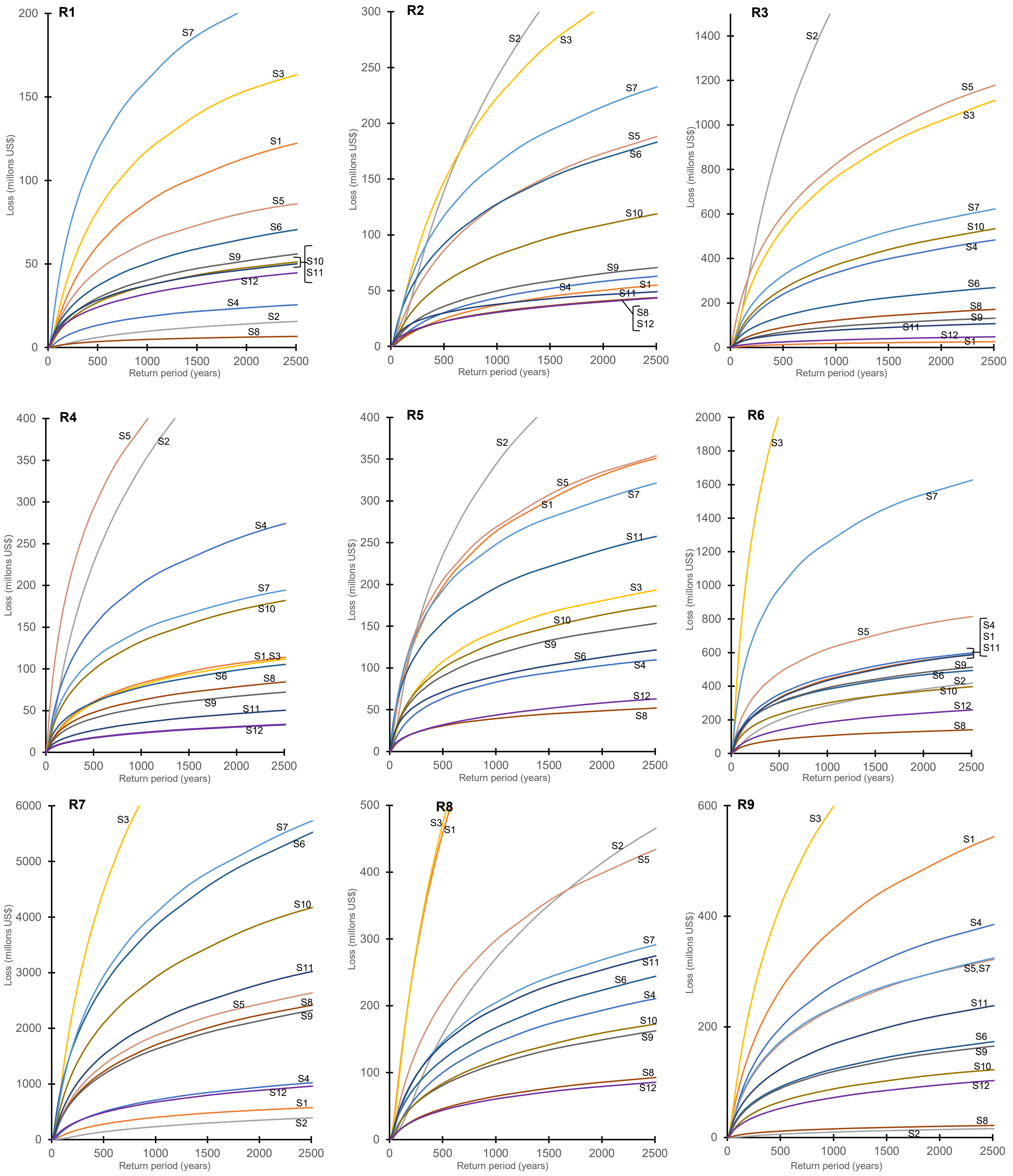
R1
Loss (millons US$)
Return period (years)
S7
S3
S1
S5
S6
S9
S10
S11
S12
S4
S2
S8
R2
S2
S3
S7
S5
S6
S10
S9
S4
S1
S11
S8
S12
R3
S2
S5
S3
S7
S10
S4
S6
S8
S9
S11
S12
S1
R4
S5
S2
S4
S7
S10
S1,S3
S6
S8
S9
S11
S12
R5
S2
S5
S1
S7
S11
S3
S10
S9
S6
S4
S12
S8
R6
S3
S7
S5
S4
S1
S11
S9
S6
S2
S10
S12
S8
R7
S3
S7
S6
S10
S11
S5
S8
S9
S4
S12
S1
S2
R8
S3
S1
S2
S5
S7
S11
S6
S4
S10
S9
S8
S12
R9
S3
S1
S4
S5,S7
S11
S6
S9
S10
S12
S8
S2

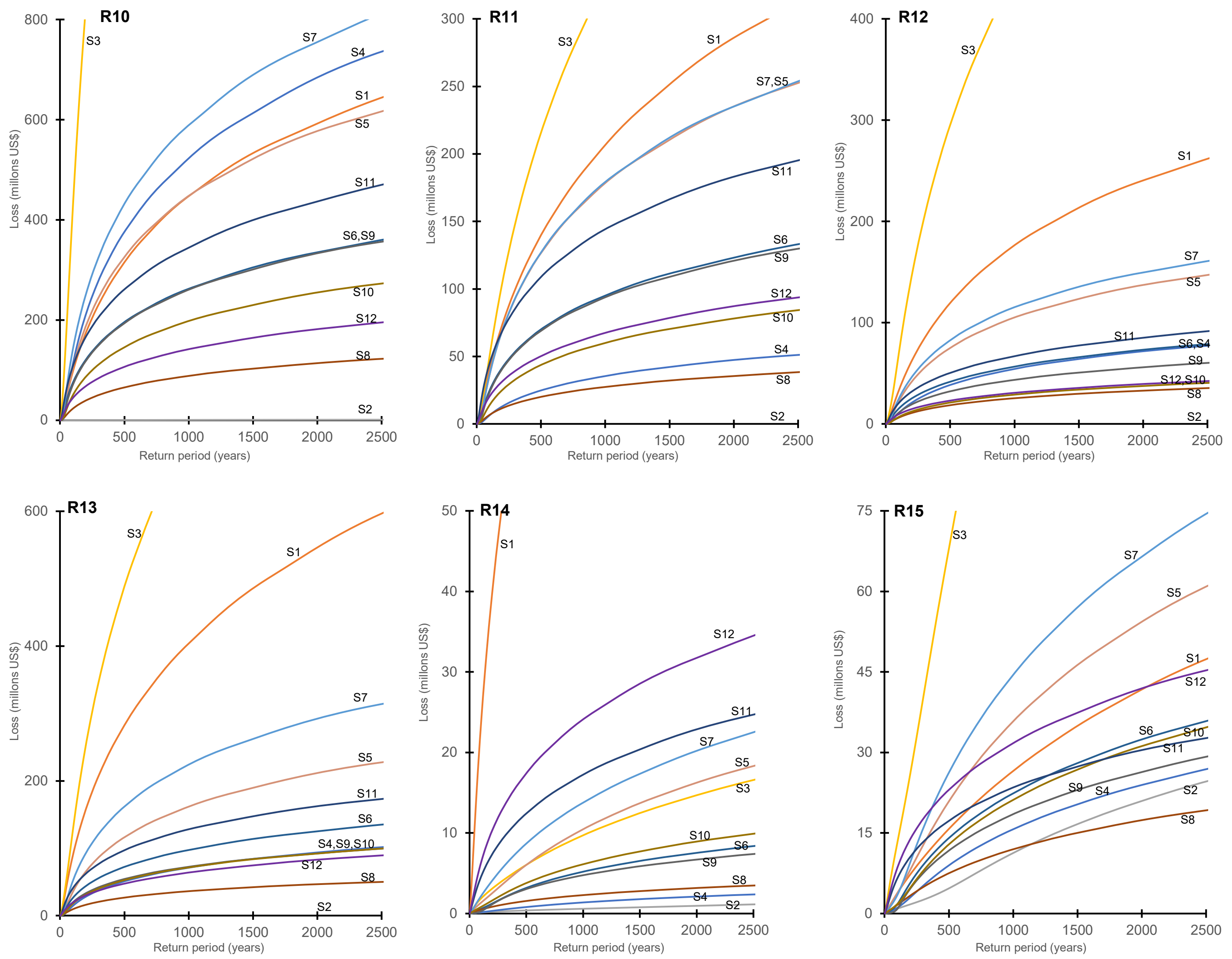

Supplementary Fig. 4 Loss exceedance curves by economic sector for each region of Chile. The loss exceedance curves are presented with the return period (the inverse of the exceedance rate) in the horizontal axis and the loss values in the vertical axis.

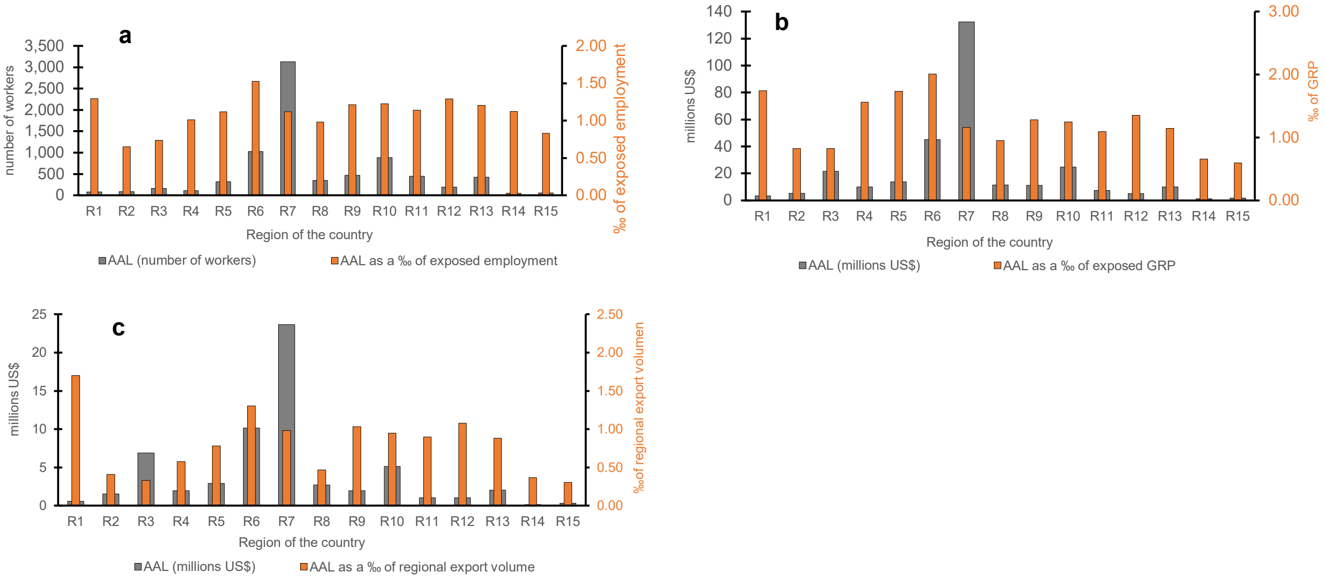

Supplementary Fig. 5 New risk indicators for Chile by region. Panel **a** shows the AAL of employment in number of workers (grey) and as a fraction (per thousand) of its total regional employment (orange). Panel **b** shows the AAL of Gross Regional Product (GRP) in million dollars (grey) and in relative terms (orange). Panel **c** shows the AAL of export volume in million dollars (grey) and as a fraction (per thousand) of its regional yearly export volume. AAL is presented as a percentage of its corresponding total value to see the influence of each region in the total AAL and as a fraction (per thousand) of its corresponding exposed value to see how risky each region is.

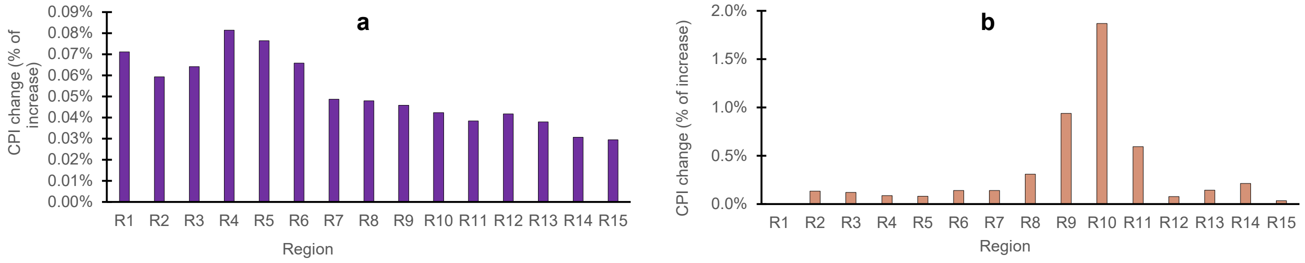

Supplementary Fig. 6 Consumer price index (CPI) change. Panel **a** illustrates the average annual increment of CPI by region of the country. Panel **b** illustrates the increment of CPI by region given the occurrence of a simulated event (Mw8.8 Maule Earthquake 2010).

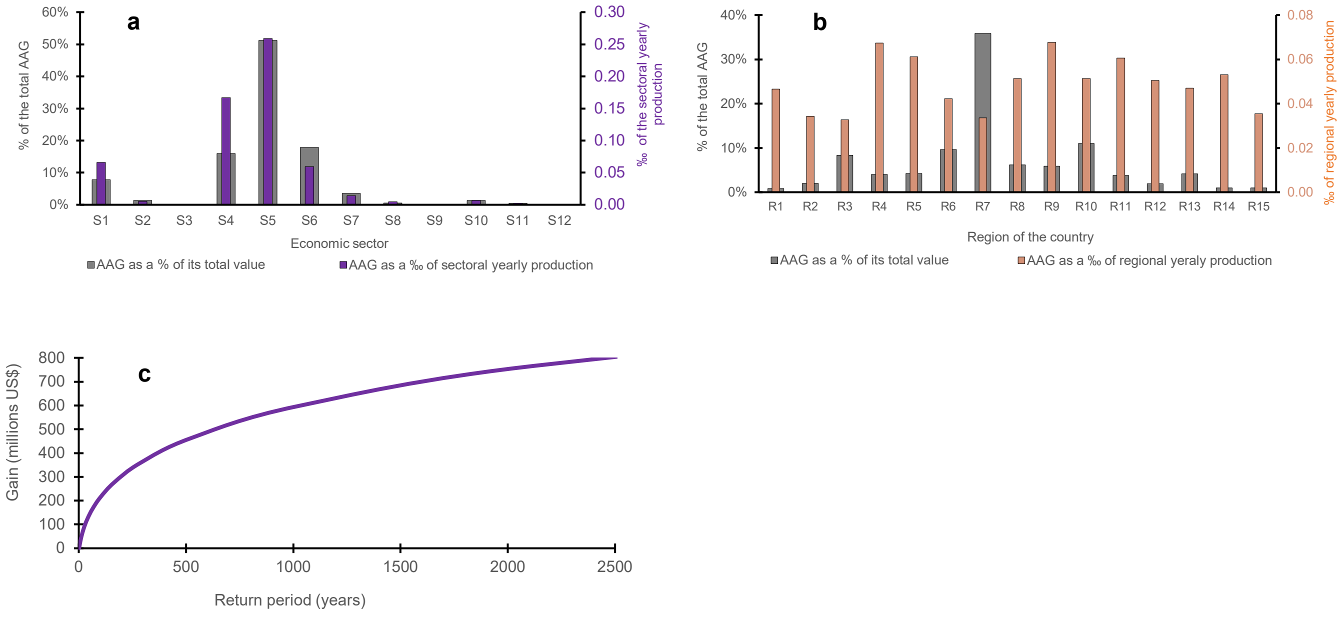

Supplementary Fig. 7 Positive economic effects in Chile. Panel a shows the average annual gain of production by sector, 1) as a percentage of its total value (gray) and 2) as a fraction (per thousand) of its total sectorial yearly production (purple). Panel b shows the average annual gain of production by region, and Panel c the gain exceedance curve of production of Chile.

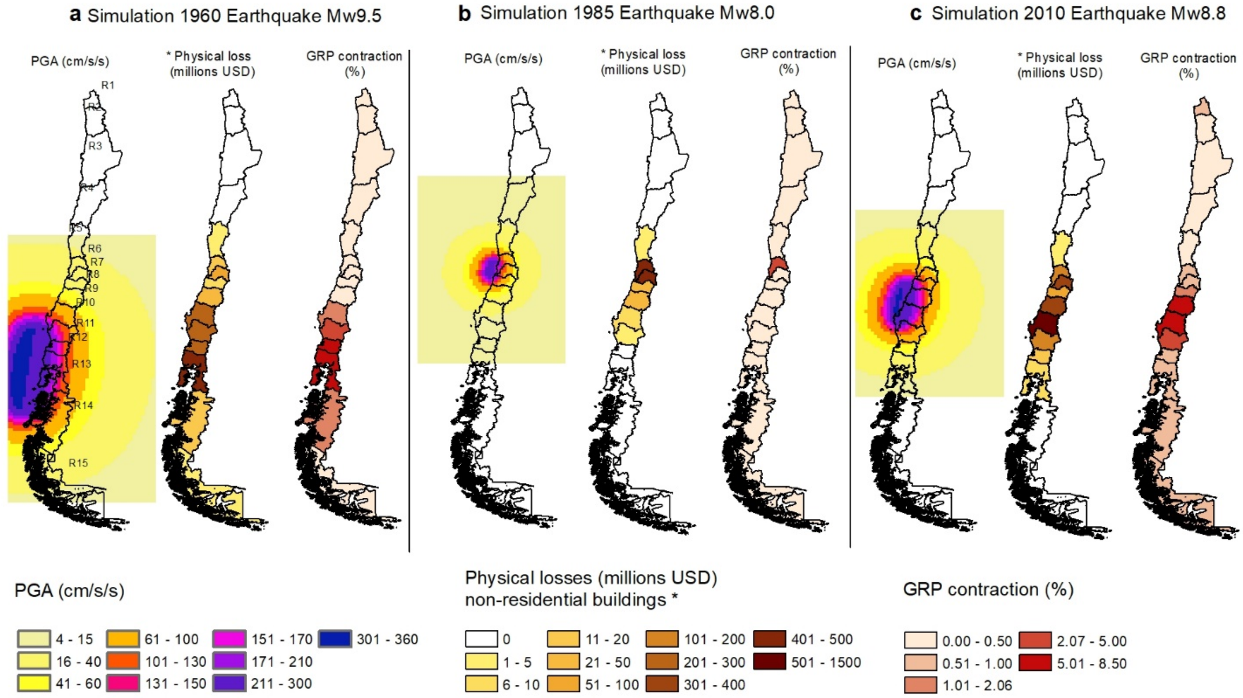

Supplementary Fig. 8 Physical losses and Gross Regional Product (GRP) contraction obtained for three simulated earthquake scenarios in Chile. Each panel presents the PGA intensity field generated by the earthquake, the physical losses in non-residential buildings and the Gross Regional Product (GRP) contraction that caused the corresponding earthquake. Panel **a** simulates the occurrence of an earthquake with similar characteristics to the 1960 Mw9.5 Valdivia Earthquake**,** Panel **b** simulates the occurrence of an earthquake with similar characteristic to the 1985 Mw8.0 Valparaiso Earthquake**,** and Panel **c** an earthquake with similar characteristic to the 2010 Mw8.8 Maule Earthquake.

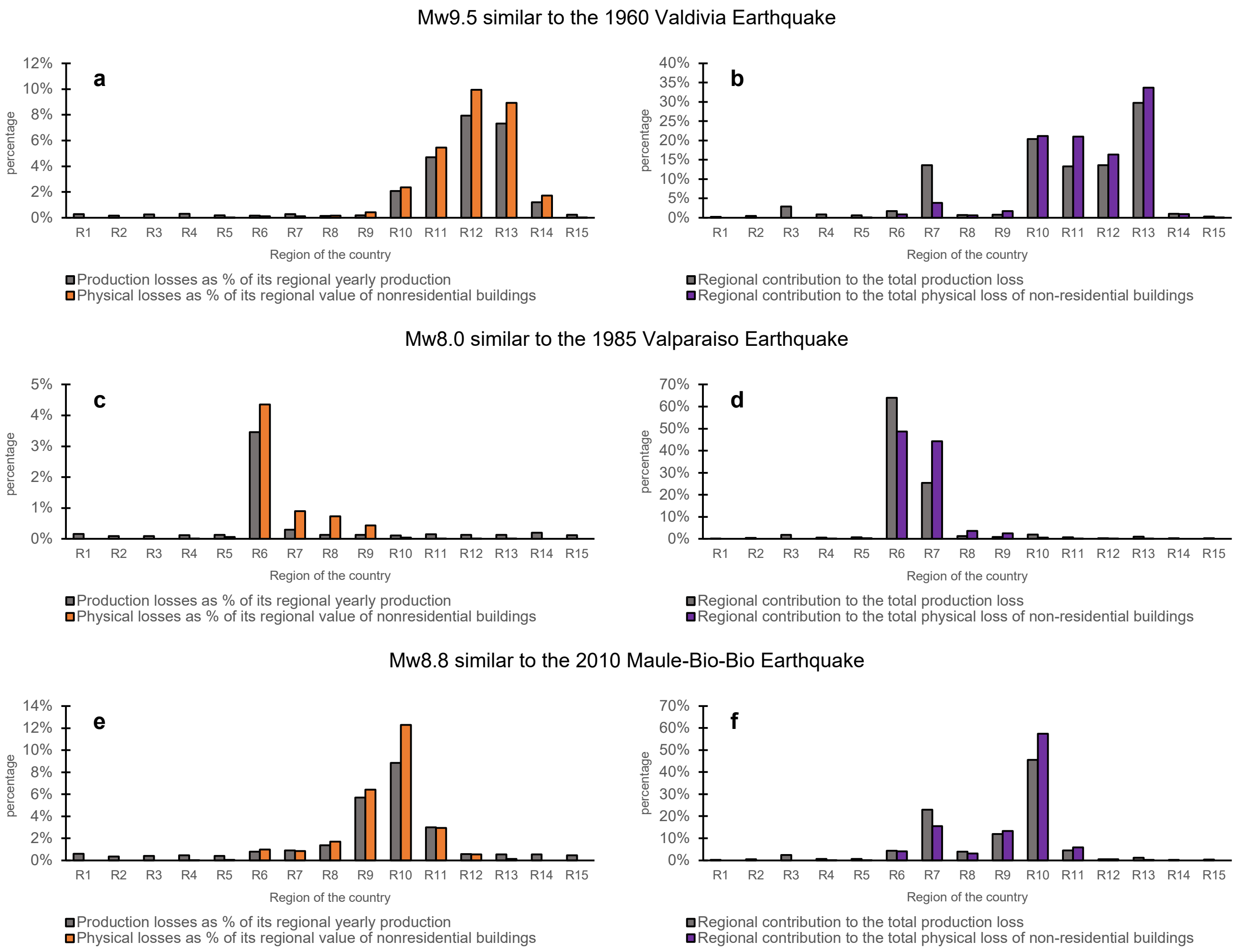

Supplementary Fig. 9 Economic losses obtained for three simulated earthquake scenarios in Chile. Panels **a, c,** and **e** show the production and physical losses caused by the earthquakes as a percentage of their corresponding total regional productions (gray bars) and their regional values of non-residential buildings (orange bars). Panels **b, d,** and **f** show the contribution of each region to the total production loss (grey bars) and the contribution of each region to the total physical loss of non-residential buildings (purple bars) caused by the earthquakes. Panels **a** y **b** present the results given the occurrence of an earthquake with similar characteristics than the 1960 Mw9.5 Valdivia Earthquake**,** Panels **c** and **d** present the results given the occurrence of an earthquake with similar characteristic than the 1985 Mw8.0 Valparaiso Earthquake**,** and Panels **e** and **f** present the loss results given the occurrence of an earthquake with similar characteristic than the 2010 Mw8.8 Maule Earthquake.

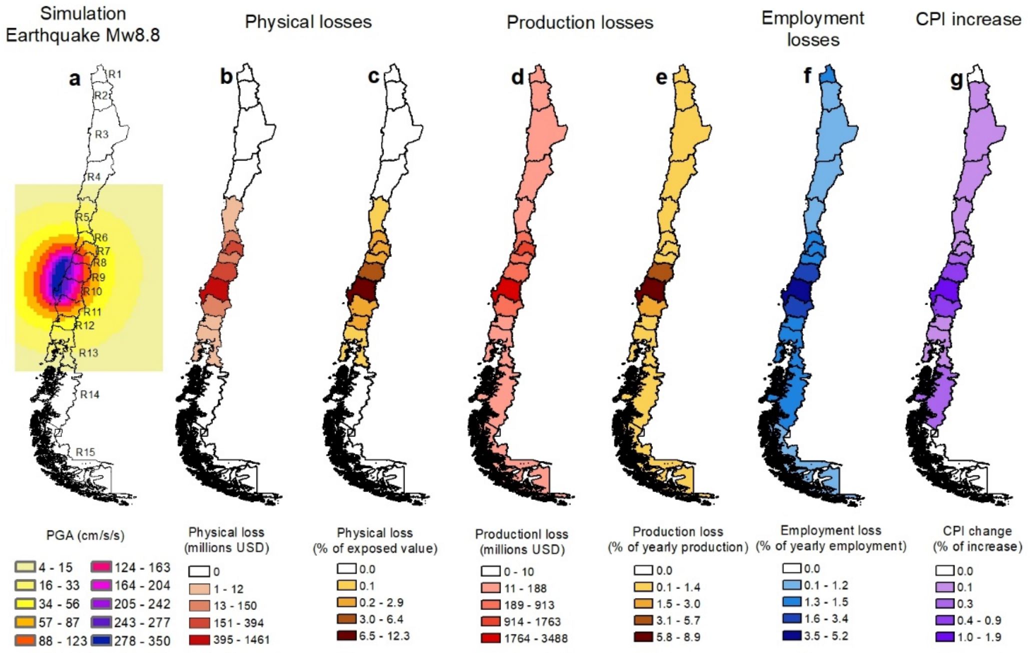

Supplementary Fig. 10 Economic losses caused in Chile by a simulated earthquake Mw8.8. Panel **a** shows the seismic intensity of the event expressed in terms of the peak ground acceleration (PGA). The average loss due to physical damage of non-residential buildings by region is shown in panel **b in** million dollars and in panel **c** as a percentage of its regional exposed value. The average loss in production by region is shown in panel **d** in millions of USD and in panel **e** as a percentage of the total annual production. Finally, panel **f** exposes the average employment loss as a percent of its corresponding regional employment, and panel **g** presents the average percentage increase of the consumer price index (CPI) by region of Chile.

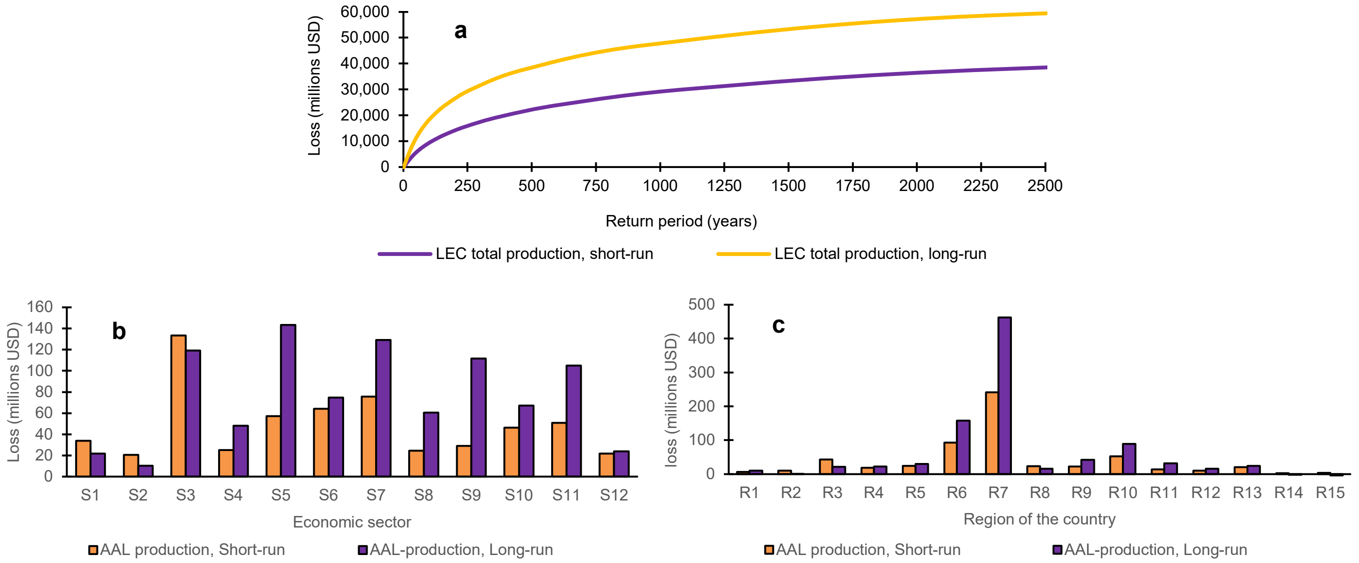

Supplementary Fig. 11 Comparison of Chile's production risk metrics associated with earthquakes occurrence and obtained for two different economic environments: short-run and long-run. Panel **a** compares the loss exceedance curves of production at the national level. Panel **b** compares the annual average losses aggregated at the sectoral level, and Panel c comparing the annual average losses aggregated at the regional level.

Supplementary Note 1 – Return period in the context of catastrophe models

In catastrophic risk modeling, it is customary to indicate the likelihood of an event taking place in, say, the following year by using its return period. In this context, the *return period* of any given loss value, *l*, is the average time between events that produce losses equal to or greater than *l*. Therefore, if a loss value has a return period of 100,000 years, this means that events that produce losses greater or equal than the given value occur, on average, every 100,000 years. Nevertheless, this does not mean that the event will take place 100,000 years in the future from now. It just means that the likelihood of this event taking place in the next year is very low. How low? Since we are considering a Poisson occurrence process in time, the annual probability of exceedance, *Pe*, and the return period, *T,* are related through:

$$Pe = 1 - e^{-\frac{1}{T}}$$

For large values of *T*,

$$Pe = \frac{1}{T}$$

Therefore, events with large return periods are not events that will occur far away in the future; they are simply unlikely events. Another heuristic way to see our simulation scheme is thinking that, in reality, we are not simulating the next 100,000 years but 100,000 times the following year.

Supplementary Note 2 – Specification of the Chilean Seismic Risk Model

This supplementary note presents the methodology applied to develop the seismic risk model of physical losses for Chile. Currently, seismic risk modeling is the standard process for estimating losses arising from earthquakes. The models are formed by a set of modules known as hazard, exposure, vulnerability, and risk (ERN-AL 2010, Cardona et al., 2012). The purpose of the models is to compute the loss exceedance curve, which indicates the annual frequency with which a given loss value is exceeded. Contemporary seismic risk models treat the most critical variables as probabilistic, so, in essence, seismic risk modeling is a procedure to rationally incorporate into the calculations the main uncertainties of the loss occurrence process. We will present highlights and details of the main modules that conform to a seismic risk model in the following lines.

The need for a seismic risk model

It is impossible to determine the loss exceedance curve on a purely empirical basis since destructive earthquakes are infrequent and exposed assets change with time. Therefore, it is not possible to construct empirical databases that allow for a reasonable empirical estimation. Because of this, the annual frequency with which given loss values are exceeded has to be estimated by indirect means, as we will describe in the following paragraphs.

Seismic hazard module

This module aims to describe, in probabilistic terms, the earthquake occurrence process. The hazard module aims to have probability distributions of the hypocentral locations of future earthquakes, their magnitudes and other source characteristics, and their occurrence times. Usually, this is expressed as an *event set*, which contains an extensive collection of individual future events, each associated with an annual probability of occurrence and to an intensity field. This intensity field also gives the spatial distribution of the local intensities induced by the earthquake in probabilistic terms. State-of-the-art seismic risk models use ground and spectral accelerations as intensity measures, and, usually, given that an event of known characteristics took place, intensities are regarded as lognormal random variables. Because of this, intensity fields usually give the spatial distribution of median and log standard deviation of the intensity measures.

Construction of events sets is part of a Probabilistic Seismic Hazard Analysis (PSHA), a methodology based on the Esteva-Cornell approach (Esteva, 1967, 1970; Cornell, 1968). This technique can attain different levels of sophistication, but a standard approach used in the field consists of the following items:

1) The territory under study is divided into *seismogenic zones*, within which it is assumed that a uniform stochastic earthquake occurrence process is taking place; the process is regarded as uniform in space (every point of the seismogenic zone has the same chance of becoming a future hypocenter) and stationary in time (probability distributions of relevant variables do not change in time). Our model defines 27 interplate, 17 intraplate, and five crustal zones, as shown in Supplementary Fig. 12.
2) Coherent with observed seismic activity and tectonic setting, each seismogenic zone is assigned a magnitude-frequency relation which specifies how often, on average, earthquakes of given magnitudes will take place. In our case, the seismic occurrence parameters of the zones were computed based on NEIC-USGS and CERESIS earthquake catalogs (CERESIS, 1997). Usually, it is assumed that the occurrence of earthquakes in time is Poissonian, so the probability distribution of time between events is exponential.
3) A ground motion prediction equation (GMPE) is assigned to the earthquakes in each seismogenic zone. GMPE's are parametric functions that relate, in probabilistic terms, earthquake source characteristics, such as magnitude, location, the orientation of the rupture plane, with the intensities experienced at arbitrary computation sites. Usually, as mentioned above, GMPE provides, for given earthquake source characteristics and site of computation, the median and the log standard deviation of the intensity, assumed to be lognormally distributed. For Chile, interplate and intraplate seismic sources were assigned the GMPE given by Youngs et al. (1997), while for crustal sources, the GMPE suggested by Sadigh et al. (1997) was used.

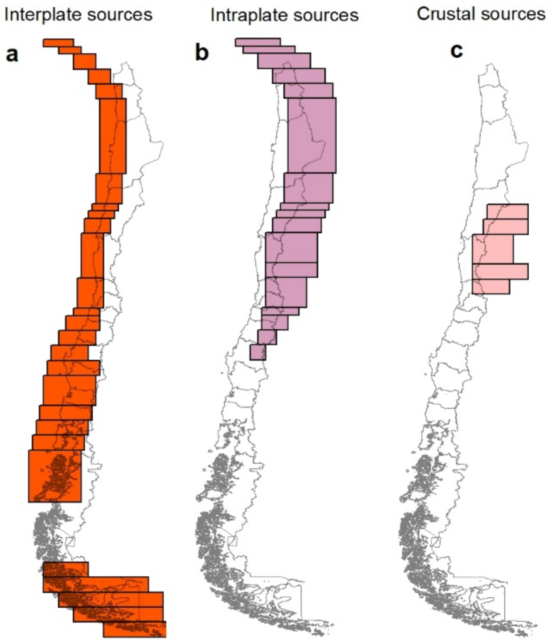

Supplementary Fig. 12 Seismic sources of Chile. Panels **a**, **b** and **c** show respectively 27 interplate seismic sources, 17 intraplate seismic sources, and five crustal seismic sources used to model the seismic hazard of Chile.

From the extensive collection of future earthquakes, each associated with a frequency of occurrence and to an intensity field, it is possible to compute the seismic hazard as a sub-product of the event set, harnessing the characteristic of mutually exclusivity and collectively exhaustively of the event set. The procedure consists of probabilistically adding up each simulated event's contribution to the seismic hazard of any particular site. The computation of the exceedance rate curves of intensities can be done with the following expression:

$$\nu(a) = \sum_{k=1}^{Events} \Pr(A_k > a)\, F_k \tag{1}$$

where $v(a)$ is the exceedance rate of intensity a, $Pr(A_k > a)$ is the probability that event k generates an intensity A in the site of interest that exceeds a, and F_k is the annual frequency of occurrence of event k. Thus, Equation 1 is one of the multiple forms that the *total probability theorem* can have.

Supplementary Fig. 13 shows results of the seismic hazard model for Chile presented using hazard maps.

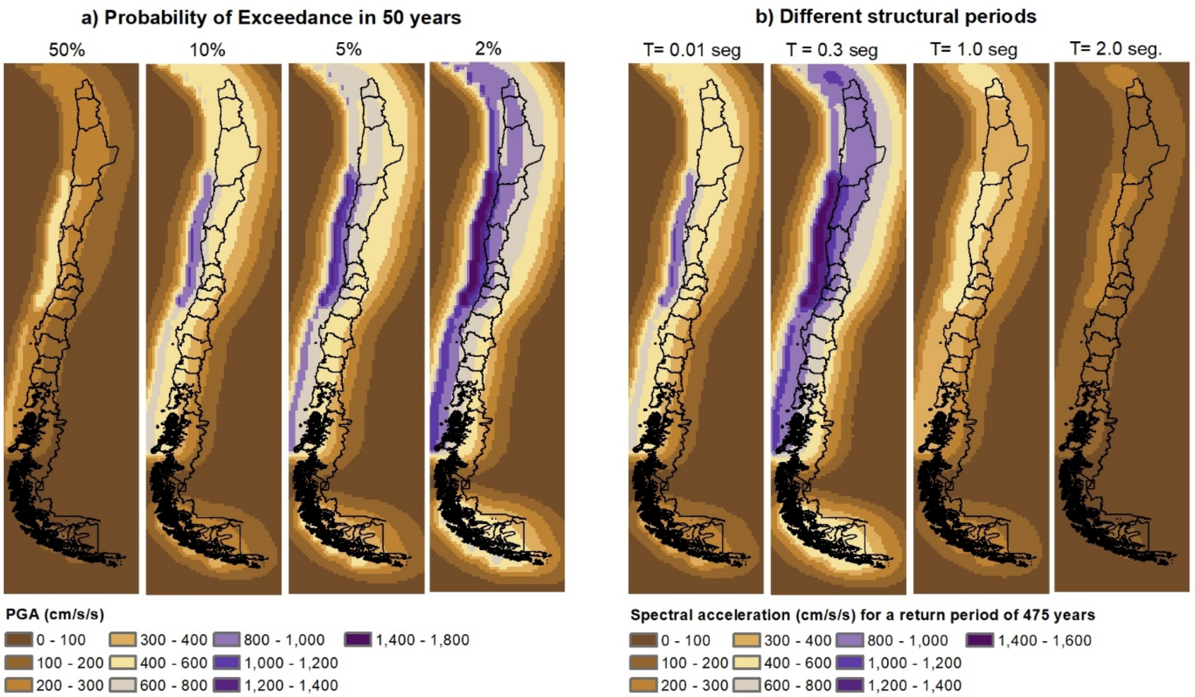

Supplementary Fig. 13 Seismic hazard results obtained for Chile. Panel **a** shows the peak ground motion acceleration (PGA) for different probabilities of exceedance in a time window of 50 years. Panel **b** shows the spectral acceleration for different structural periods computed for a return period of 475 years (10% of probability of exceedance in 50 years).

Exposure module

The exposure component of a classical risk model gives information about the location, characteristics, and value of all the relevant assets for the risk analysis; in general, the database should include all assets susceptible to experiencing damage during earthquakes. Three components are indispensable in a classical exposure model:

1) Geo-location of the assets. Location is relevant since damage during a given earthquake depends on the relative location of the asset concerning the hypocenter; in general, the closer to the hypocenter, the more significant the loss.

2) Vulnerability information. As we will see later, the vulnerability of an asset defines the intensity-loss relations; therefore, some structural characteristics are required to establish these relations adequately. In the case of buildings, the relevant characteristics are age, structural systems, construction type, number of floors, predominant use, and wall and roof materials.

3) Replacement cost and human occupancy of assets. This information is required to give appropriate scale to the direct losses inflicted by the earthquake to the assets.

While these are the three components of the exposure component of a classical risk model, in which only direct losses are of interest, in our case, we need to include a fourth component, which is the economic group or sector to which each asset belongs. This is needed because, in our approach to evaluating indirect losses, the direct losses are used to indicate the size of the stock capital decreases in a given sector, so we need to know to which sector the asset belongs.

In our case of study, the database of non-residential buildings of Chile was estimated using a methodology developed by Gunasekera et al. (2015), Aubrecht and León (2015, 2016), Pomonis (2014), which harnesses global geo-located datasets and census information to generate a proxy building exposure model of an entire country or region (León, 2019). Three key components conform to the methodology: 1) exposure disaggregation, 2) building stock estimate, and 3) assets cost estimate.

The exposure disaggregation process uses global geo-located datasets to estimate the population density distribution within a specific region. An essential assumption is that the population density is a proxy of the housing density, and in general, a proxy of the density of built-up areas (Aubrecht et al., 2013), (Aubrecht et al., 2014). Thus, estimating the population density allows us to estimate the housing density distribution. Several global datasets are openly available (e.g., Gunasekera et al., 2015) helpful for this disaggregation process; in our case, we used LandScan, Global Human Settlement (GHS-POP), WorldPop, and VIIRS.

LandScan (https://landscan.ornl.gov/), GHS-POP (https://ghsl.jrc.ec.europa.eu/), and WoldPop (https://www.worldpop.org/) are datasets which provide the distribution and density of the global population at different resolution levels, by combining spatial data mining technologies and multivariate dasymetric modeling techniques with demographic, geographic and remote sensing data. VIIRS sensor onboard the Suomi NPP satellite platform provides information of dim nighttime scenes worldwide with a resolution of 15 arc-sec spatial resolution (https://earthdata.nasa.gov/earth-observation-data/near-real-time/download-nrt-data/viirs-nrt). Radiance values of nighttime scenes can be related to the presence of built-up areas, as we explain later.

The first three layers are used to estimate the spatial density distribution of the Chilean population based on a weighted average process that considers the three layers' contribution. The VIIRS dataset is used to discriminate which population cells are considered non-residential usage (Aubrecht & León 2016); the higher the VIIRS value, the higher the probability of being a non-residential cell. The procedure is applied to each region of Chile. We focused our attention exclusively on non-residential buildings because we assume that the damage in non-residential buildings is direct and highly correlated with the reduction of the capital stock available to the production process of direct industries and sectors of the economy. As a result of the disaggregation process, we obtain the non-residential population distribution by region of Chile with a resolution of 30 arc-second grid cells (approx. 1 km^2). Supplementary Figure 14 exemplifies the global datasets used in the disaggregation process for two regions of Chile.

In the second component, we estimate the non-residential building inventory of Chile by using census and local information about the structural features of buildings. First, we define a typical building type by each economic sector of Chile, assuming that a building type well represents the structural behavior of buildings in a sector. This information will be useful to generate and calibrate the vulnerability functions used in the following subsection. Furthermore, we estimate the built floor area (m^2) by sector/region of Chile, for instance, the built floor area of buildings of the manufacturing industry (S3) in the Metropolitan Region of Santiago (R7). Finally, we used the national employment survey of Chile to obtain the employment distribution by sector/region of Chile, and then, we multiplied the number of workers by sector/region by an average built floor area per worker. In our case, built floor areas per worker for different building occupancies (León, 2019) were computed based on data provided by Reinoso et al. (2012) and Alvarez et al. (2015).

To estimate the cost or the replacement value of assets by sector/region required in the third component of the methodology, we multiply the built floor area by sector/region by an average construction cost per square meter of non-residential buildings in Chile. In our case, we adopted the construction cost of the non-residential building proposed by the SARA project (Alvarez, 2015).

Finally, we distribute the total construction cost of each economic sector/region according to the non-residential population distribution of its corresponding region to generate a set of layers of 30 arc-second grid cells (approx. 1 km2) with all necessary information to carry out a traditional seismic risk assessment. For instance, Supplementary Fig. 15 presents the geographic distribution of Chile's total exposure value of non-residential buildings.

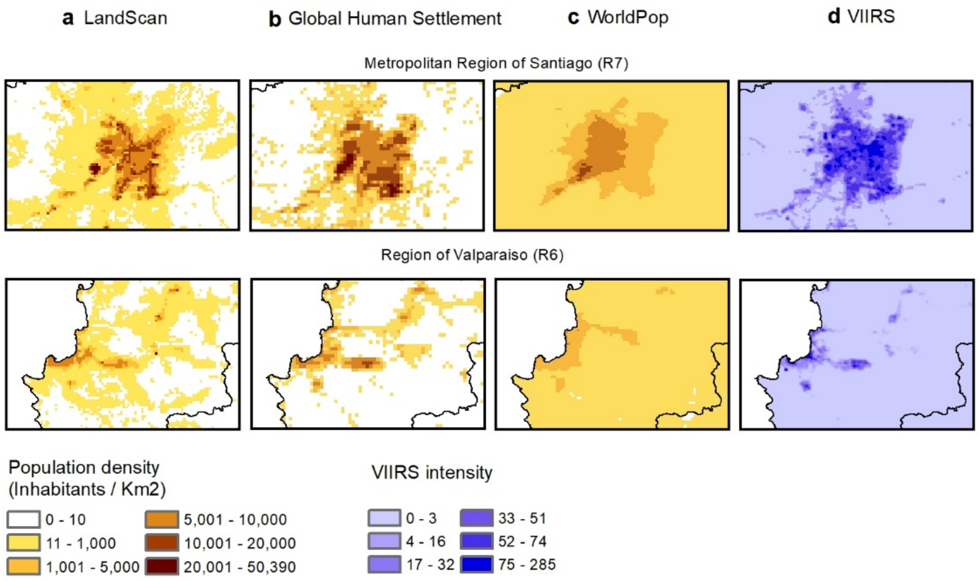

Supplementary Fig. 14 Global datasets used to estimate non-residential building inventory of Chile. Panels **a**, **b**, **c,** and **d** present respectively LandScan2017, Global Human Settlement 2015, WordPop 2015, and VIIRS 2016 datasets for two regions of Chile used in the disaggregation process to estimate non-residential population distribution.

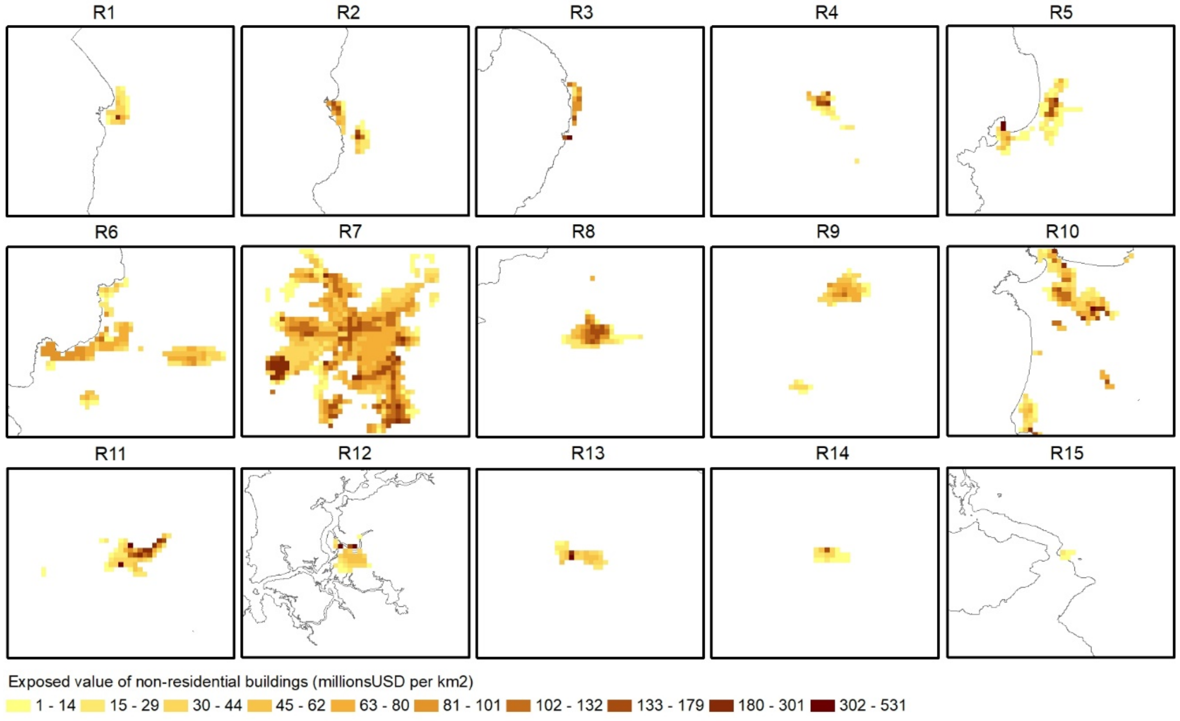

Supplementary Fig. 15 Exposure model of non-residential buildings of Chile by region. The figure illustrates the total exposed economic value at 30 arc-second spatial resolution (approximately 1 x 1-km grid at the equator) in million US dollars for the most densely built part of each region.

Vulnerability module

Vulnerability is defined as the predisposition of a system, element, or any component, to be affected in the face of a specific peril occurrence. Thus, it is possible to determine the vulnerability of buildings and any asset susceptible to be damaged. In our case, relevant assets are buildings, factories, infrastructure, and, in general, all assets whose damage might have a potential impact on the economic flows.

The vulnerability is different from one asset to another, and it changes according to the hazard type and its structural characteristics. For instance, buildings in a region might be very vulnerable to hurricanes but not vulnerable to earthquakes. Initially developed for the structural engineering field, vulnerability functions (Ordaz M. , 2000), ERN-AL (2010) establish probabilistic relations between the intensity of the seismic motion and the loss caused in the structure when an event of such intensity takes place. Losses are treated as random variables, so usually, a vulnerability function relates intensity with the mean and the standard deviation of the losses as a function of the seismic demand. Supplementary Fig. 16 presents the vulnerability functions used for non-residential buildings of Chile, as reported in Leon (2021).

It is common to assume that the loss for a given intensity, expressed as a fraction of the replacement value, is Beta distributed. Thus, vulnerability functions as those of Supplementary Figure 9 give the first two moments of the loss as a function of intensity. Since the loss is assumed to be Beta distributed, these two moments suffice to determine the probability distribution of the loss given an intensity value.

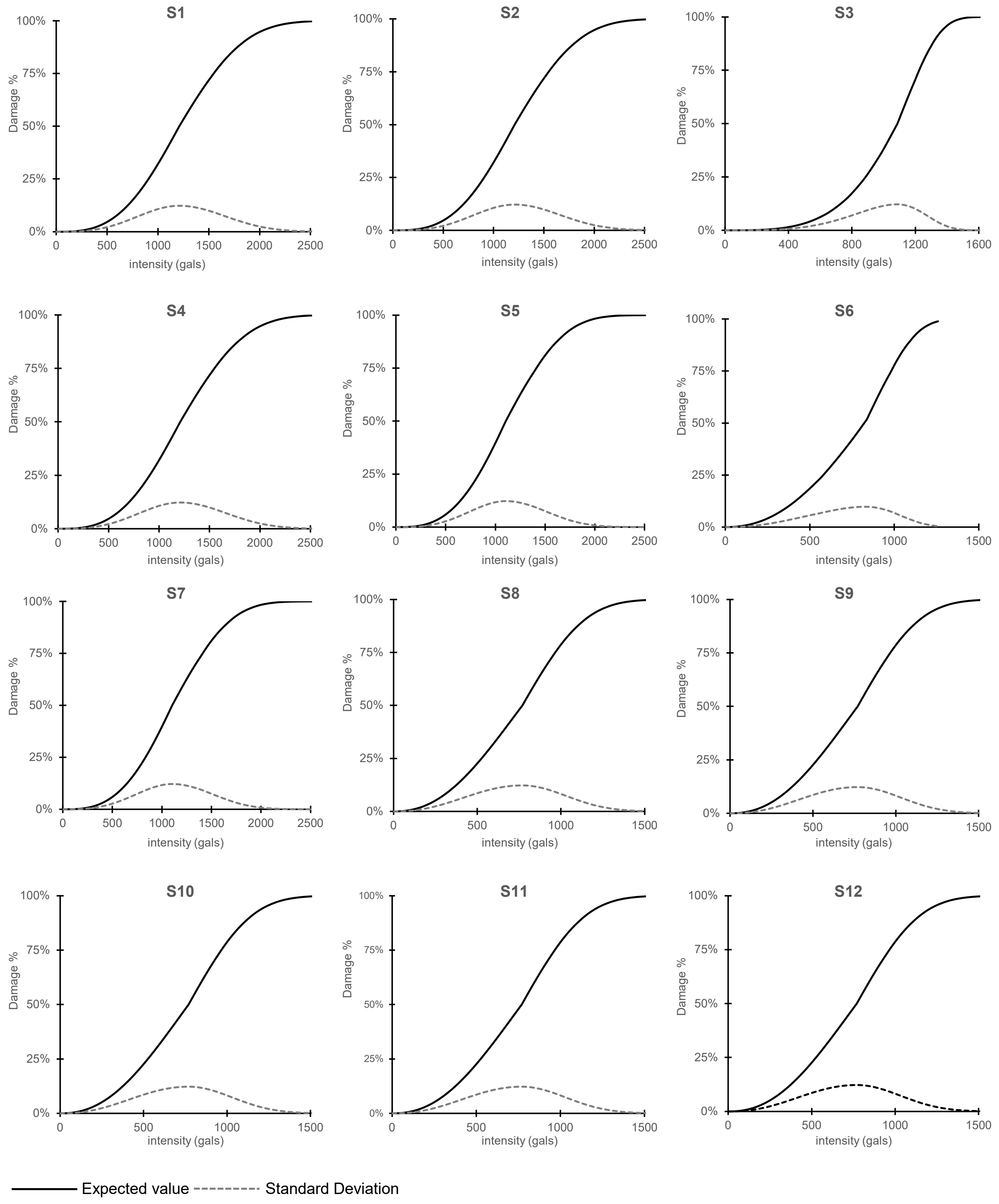

Supplementary Fig. 16 Vulnerability functions used for Chile. They refer to physical damage of non-residential buildings that made up the capital stock of economic sectors S1-S12. In this case, the intensity is given in terms of the spectral acceleration. Seismic vulnerability functions set up relations between the intensity of the seismic motion and the loss caused in the structure when an event of such intensity takes place.

Risk module

The traditional seismic risk assessment of physical losses is carried out by way of putting together information related to hazard, exposed assets, and vulnerability through equations 1 and 3, presented in the main manuscript, which compute the standard metrics used in the disaster risk literature: the average annual loss and the loss exceedance curve, handling and propagating the uncertainties involved in the process.

In equations 1 and 3, the value of the loss Ld_k is the summation of the losses suffered by assets affected by earthquake *k*. Depending on the loss's aggregation level, the summation can be done for the entire country, sector, region, or sector/region. For instance, we can compute the loss suffered by non-residential buildings belonging to the manufacturing sector and located in the Region of Valparaiso. Note that individual losses caused by a particular earthquake are geographically dispersed along the affected area and will have a certain level of correlation. In addition, individual losses of assets to be aggregated are modeled as random variables (Beta distributed), of which we know their corresponding probability distributions. At this point, it is assumed that the aggregated loss Ld_k is a new random variable with Beta distribution whose two first statistic moments, $E(Ld_k)$ and $VAR(Ld_k)$, can be obtained with the following expressions:

$$E(Ld_k) = \sum_j E(L_j)$$

$$VAR(Ld_k) = \sum_j VAR(L_j) + \sum_j \sum_i \rho \sqrt{VAR(L_j)VAR(L_i)} \qquad j \neq i$$

where $E(L_j)$ and VAR(L_j) are the expected value and the standard deviation of the loss of the asset *j* caused by earthquake *k*, while ρ is the correlation coefficient between pairs of losses. Unfortunately, there is no universally accepted methodology to compute the correlation coefficients, and it is not possible to estimate them by purely empirical means. Therefore, we have resorted to simulations and sensitivity analysis in order to establish reasonable values for these coefficients based on two main criteria: 1) the increment on the variance of the aggregated loss must not be excessively large, and 2) the effect of aggregating an extensive portfolio of buildings must be noticeable but not too large such that the variance maintains a reasonable level of uncertainty in *Ld*.

Finally, and once $E(Ld_k)$ and VAR(Ld_k) have been computed, to have defined entirely the probability distribution of the loss $Ld_{k,}$ it is required to define the range of the Beta distribution. The range is computed as the summation of the value of all assets that have some chance of being damaged by earthquake *k*. Note that the election of the maximum value of the loss Ld has influence in its probability distribution and thus on the risk results; we know these effects as the Lionel paradox (León, 2021). After this, we have everything we need to calculate the probability that direct losses, *Ld,* exceed a given value *l*, $Pr(Ld > l)$, given the occurrence of earthquake *k*.

Supplementary Note 3 - Loss aggregation process

In general, and because of the lower geographical resolution of the CGE models compared to that of the seismic risk models, a loss aggregation is required in order to sum all the losses that correspond to the same economic sector at the same economic region. Since the losses at the various assets are not fixed numerical values but correlated random variables, the aggregation process is not trivial because of the correlation among losses for the same event. Thus, the aggregated loss L_A is a new random variable assumed with Beta distribution whose expected value $E(L_A)$ and standard deviation $VAR(L_A)$ is given by:

$$E(L_A) = \sum_j E(L_j)$$

$$VAR(L_A) = \sum_j VAR(L_j) + \sum_j \sum_k \rho \sqrt{VAR(L_j)VAR(L_k)} \qquad j \neq k$$

where $E(L_j)$ and $VAR(L_j)$ are the expected value and the standard deviation of the loss of the asset j belonging to the same economic sector at the same economic region while ρ, is the correlation coefficient between pairs of losses. At this point, we assume that the new aggregated loss is a reasonable measure of the total loss (or damage) undergone by the capital stock of a sector belonging to an economic region.

Supplementary Note 4 - Specification of the BMCH Model

This supplementary note presents the analytical, functional, and numerical structures of Chile's interregional general equilibrium model, the Chilean version of the B-MARIA[1] Chilean model (BMCH). First, the specification of the linearized form of the model is provided, based on different groups of equations. The notational convention uses uppercase letters to represent the levels of the variables and lowercase for their percentage-change representation. Thus, superscripts *(u)*, *u = 0, 1j, 2j, 3, 4, 5*, 6 refer, respectively, to output *(0)* and the six different regional-specific users of the products identified in the model[2]: producers in sector *j (1j)*, investors in sector *j (2j)*, households *(3)*, purchasers of exports *(4)*, regional governments *(5)*, and central government *(6)*; the second superscript *(r)* identifies the domestic region where the user is located. Two subscripts identify inputs: the first *(i)* takes the values *1, ..., g*, for commodities, *g + 1*, for primary factors; the second subscript identifies the source of the input, being it from domestic region *b (1b)* or imported *(2)*, or coming from labor *(1)* or capital *(2)*, the two primary factors in the model. The symbol (•) is employed to indicate a sum over an index.

We define the following sets: $G = \{1, \dots, g\}$, where g is the number of composite goods; $G^* = \{1, \dots, g, g+1\}$, where g+1 is the number of composite goods and primary factors, with $G^* \supset G$; $H = \{1, \dots, h\}$, where h is the number of industries; $U = \{(3), (4b), (5), (6), (kj)\}$ for $k = (1), (2)$ and $j \in H$, is the set of all users in the model; $U^* = \{(3), (5), (6), (kj)\}$ for $k = (1), (2)$ and $j \in H$, with $U \supset U^*$, is the subset of domestic users; $S = \{1, \dots, r, r+1\}$, where r+1 is the number of all regions (including foreign); $S^* = \{1, \dots, r\}$, with $S \supset S^*$, is the subset with the r domestic regions; and $F = \{1, \dots, f\}$ is the set of primary factors. In the BMCH model, $g = h = 12$, $r = 15$, and $f = 2$.

We model the sourcing of composite goods based on multilevel structures, which enable a significant number of substitution possibilities. We employ nested sourcing functions to create composite goods available for consumption in the regions of the model. We assume that domestic users, i.e., firms, investors, households, and government, use combinations of composite goods specified within two-level CES nests. At the bottom level, bundles of domestically produced goods are formed as combinations of goods from different regional sources. At the top level, substitution is possible between domestically produced and imported goods. Equations (A1) and (A2) describe the regional sourcing of domestic goods and the substitution between domestic and imported products.

$$x_{(i(1b))}^{(u)r} = x_{(i(1\bullet))}^{(u)r} - \sigma 1_{(i)}^{(u)r} \left(p_{(i(1b))}^{(u)r} - \sum_{l \in S^*} \left(\frac{V(i,1l,(u),r)}{V(i,1\bullet,(u),r)} \right) \left(p_{(i(1l))}^{(u)r} \right) \right)$$
$$i \in G;\ b \in S^*; (u) \in U^*;\ r \in S^* \qquad \text{(A1)}$$

where $x_{(i(1b))}^{(u)r}$ is the demand by user *(u)* in region r for good i in the domestic region *(1b)*; $p_{(i(1b))}^{(u)r}$ is the price paid by user *(u)* in region r for good i in the domestic region *(1b)*; $\sigma 1_{(i)}^{(u)r}$ is a parameter measuring the user-specific elasticity of substitution between

[1] B-MARIA is the Brazilian Multisectoral and Regional-Interregional Analysis Model

[2] We have specified a seventh residual user, *(7)*, to deal with statistical discrepancies in the balancing of the model's absorption matrix based on the Chilean interregional input-output system (IIOS).

alternative domestic sources of commodity *i*, known as the regional trade Armington elasticity; and $V(i, 1l, (u), r)$ is an input-output flow coefficient that measures purchasers' value of good *i* from domestic source *l* used by user *(u)* in region *r*.

$$x_{(is)}^{(u)r} = x_{(i\bullet)}^{(u)r} - \sigma 2_{(i)}^{(u)r} \left(p_{(is)}^{(u)r} - \sum_{l=1\bullet,2} \left(\frac{V(i,l,(u),r)}{V(i,\bullet,(u),r)} \right) \left(p_{(il)}^{(u)r} \right) \right)$$
$$i \in G;\ s = 1\bullet, 2;\ (u) \in U^*;\ r \in S^* \qquad \text{(A2)}$$

where $x_{(is)}^{(u)r}$ is the demand by user *(u)* in region *r* for either the domestic composite or the foreign good *i*; $p_{(is)}^{(u)r}$ is the price paid by user *(u)* in region *r* for either the domestic composite or the foreign good *i*; $\sigma 2_{(i)}^{(u)r}$ is a parameter measuring the user-specific elasticity of substitution between the domestic bundle and imports of good *i*, known as the international trade Armington elasticity; and $V(i, l, (u), r)$ is an input-output flow coefficient that measures purchasers' value of good *i* from either the aggregate domestic source or the foreign source *l* used by user *(u)* in region *r*.

In addition to goods used as intermediate inputs, firms in the model also demand primary factors of production. The equations that describe the industry *j*'s demands inputs are derived under the assumption of Leontief technology with Armington nests (imperfect substitution between inputs of the same type from different sources). In our specification of the nested production functions, we assume firms to use combinations of composite intermediate inputs, formed according to Equations (A1) and (A2), and primary factor composites. In the case of the primary factor bundle, substitution is possible among different types of primary factors. Equation (A3) specifies the substitution between labor and capital in the model. It is derived under the assumption that industries choose their primary factor inputs to minimize costs subject to obtaining sufficient primary factor inputs to satisfy their technological requirements (nested Leontief/CES specification). We have included technical change variables to allow for factor-specific productivity shocks. We model the combination of intermediate inputs, and the value added (primary factors) aggregate in fixed proportions at the top of the nested production function, assuming no substitution between primary factors and other inputs. The Leontief specification is presented in Equation (A4). More flexible functional forms have been rarely introduced in multi-regional models, mainly due to data availability constraints. In addition to a technical coefficient in the relation between the sectoral demand for the primary factor composite and the total output, we have included a scale parameter. This modeling procedure has been based on previous studies made by Haddad and Hewings (2005), which allows for the introduction of Marshallian agglomeration (external) economies by exploring local properties of the CES function.

$$x_{(g+1,s)}^{(1j)r} - a_{(g+1,s)}^{(1j)r} = \alpha_{(g+1,s)}^{(1j)r} x_{(g+1,\bullet)}^{(1j)r} - \sigma 3_{(g+1)}^{(1j)r} \left(p_{(g+1,s)}^{(1j)r} + a_{(g+1,s)}^{(1j)r} - \sum_{l \in F} \left(\frac{V(g+1,l,(1j),r)}{V(g+1,\bullet,(1j),r)} \right) \left(p_{(g+1,l)}^{(1j)r} + a_{(g+1,l)}^{(1j)r} \right) \right)$$
$$j \in H;\ s \in F;\ r \in S^* \qquad \text{(A3)}$$

where $x_{(g+1,s)}^{(1j)r}$ is the demand by sector *j* in region *r* for each primary factor; $a_{(g+1,s)}^{(1j)r}$ is the exogenous sector-specific variable of (saving) technical change for primary factor *s* in region *r*; $p_{(g+1,s)}^{(1j)r}$ is the price paid by sector *j* in region *r* for primary factor *s*; $\sigma 3_{(g+1)}^{(1j)r}$ is a parameter measuring the sector-specific elasticity of substitution among different primary factors; and $V(g+1, l, (1j), r)$ is an input-output flow coefficient that measures purchasers' value of factor *l* used by sector *j* in region *r*.

$$x_{(i\bullet)}^{(1j)r} = \mu_{(g+1,\bullet)}^{(1j)r} z^{(1j)r} + a_{(i)}^{(1j)r}$$
$$j \in H;\ i \in G^*;\ r \in S^* \qquad \text{(A4)}$$

where $x_{(i\bullet)}^{(1j)r}$ is the demand by sector *j* in region *r* for the bundles of composite intermediate inputs and primary factors *i*; $z^{(1j)r}$ is the total output of sector *j* in region *r*; $a_{(i)}^{(1j)r}$ is the exogenous sector-specific variable of technical change for composite intermediate inputs and primary factors in region *r*; and $\mu_{(i\bullet)}^{(1j)r}$ is a scale parameter measuring the sector-specific returns to the composite of primary factors in each region.

Units of capital stock are created for industry *j* at minimum cost. Commodities are combined via a Leontief function, as specified in Equation (A5). As described in Equations (A1) and (A2), regional, and domestic, and imported commodities are combined, respectively, via a CES specification (Armington assumption). No primary factors are used in capital creation. The use of these inputs is recognized through the capital goods-producing sectors in the model, mainly machinery and equipment industries, construction, and support services.

$$x_{(i\bullet)}^{(2j)r} = z^{(2j)r} + a_{(i)}^{(2j)r}$$
$$j \in H;\ i \in G;\ r \in S^* \qquad \text{(A5)}$$

where $x_{(i\bullet)}^{(2j)r}$ is the demand by sector *j* in region *r* for the bundles of composite capital goods *i*; $z^{(2j)r}$ is the total investment of sector *j* in region *r*; $a_{(i)}^{(2j)r}$ is the exogenous sector-specific variable of technical change for changing the composition of the sectoral unit of capital in region *r*.

In deriving the household demands for composite commodities, we assume that households in each region behave as a single, budget-constrained, utility-maximizing entity. The utility function is of the Stone-Geary or Klein-Rubin form. Equation (A6) determines the optimal composition of household demand in each region. Total regional household consumption is determined as a function of real household income. The demands for the commodity bundles in the nesting structure of household demand follow the CES pattern established in Equations (A1) and (A2), in which an activity variable and a price-substitution term play the major roles. In Equation (A6), consumption of each commodity *i* depends on two components: first, for the subsistence component, which is defined as the minimum expenditure requirement for each commodity, changes in demand are generated by changes in the number of households and tastes; second, for the luxury or supernumerary part of the expenditures in each good, demand moves with changes in the regional supernumerary expenditures, changes in tastes, and changes in the price of the composite commodity. The two components of household expenditures on the composite commodities are weighted by their respective shares in the total consumption of the composite commodity.

$$V(i,\bullet,(3),\mathrm{r})\left(p_{(i\bullet)}^{(3)r} + x_{(i\bullet)}^{(3)r} - a_{(i\bullet)}^{(3)r}\right)$$
$$= \gamma_{(i)}^{r} P_{(i\bullet)}^{(3)r} Q^{r}\left(p_{(i\bullet)}^{(3)r} + x_{(i\bullet)}^{(3)r} - a_{(i\bullet)}^{(3)r}\right) + \beta_{(i)}^{r}\left(C^{r} - \sum\nolimits_{j\in G} \gamma_{(j)}^{r} P_{(j\bullet)}^{(3)r} Q^{r}\left(p_{(j\bullet)}^{(3)r} + x_{(j\bullet)}^{(3)r} - a_{(i\bullet)}^{(3)r}\right)\right)$$
$$i \in G;\ r \in S^* \qquad \text{(A6)}$$

where $p_{(i\bullet)}^{(3)r}$ is the price paid by household in region *r* for the composite good *i*; $x_{(i\bullet)}^{(3)r}$ is the household demand in region *r* for the composite good *i*; $a_{(i\bullet)}^{(3)r}$ is the commodity-specific variable of regional taste change; Q^r is the number of households in region *r*; C^r is the total expenditure by household in region *r*, which is proportional to regional labor income; $\gamma_{(i)}^{r}$ is the subsistence parameter in the linear expenditure system for commodity *i* in region *r*; $\beta_{(i)}^{r}$ is the parameter defined for commodity *i* in region *r* measuring the marginal budget shares in the linear expenditure system; and $V(i,\bullet,(3),\mathrm{r})$ is an input-output flow coefficient that measures purchasers' value of good *i* consumed by households in region *r*.

As noted by Peter et al. (1996), a feature of the Stone-Geary utility function is that only the above-subsistence, or luxury, component of real household consumption, $utility^{(r)}$, affects the per-household utility, as described in Equation (A7).

$$utility^{(r)} = \left(C^{r} - \sum\nolimits_{j\in G} \gamma_{(j)}^{r} P_{(j\bullet)}^{(3)r} Q^{r}\left(p_{(j\bullet)}^{(3)r} + x_{(j\bullet)}^{(3)r} - a_{(i\bullet)}^{(3)r}\right)\right) - q^{r} - \sum\nolimits_{i\in G} \beta_{(i)}^{r}\, p_{(i\bullet)}^{(3)r}$$
$$r \in S^* \qquad \text{(A7)}$$

where q^r is the percentage change in the number of households in each region.

In Equation (A8), foreign demands (exports) for domestic good *i* depend on the percentage changes in a price and three shift variables which allow for vertical and horizontal movements in the demand curves. The price variable that influences export demands is the purchaser's price in foreign countries, including the relevant taxes and margins. The parameter $\eta^{r}_{(is)}$ controls the sensitivity of export demand to price changes.

$$\left(x^{(4)r}_{(is)} - fq^{(4)r}_{(is)}\right) = \eta^{r}_{(is)}\left(p^{(4)r}_{(is)} - phi - fp^{(4)r}_{(is)}\right)$$
$$i \in G;\ r, s \in S^{*} \qquad \text{(A8)}$$

where $x^{(4)r}_{(is)}$ is foreign demand for domestic good *i* produced in region *s* and sold from region *r* (in the model there are no re-exports so that *r* = *s*); $p^{(4)r}_{(is)}$ is the purchasers' price in the domestic currency of exported good *i* demand in region *r*; phi is the nominal exchange rate; and $fq^{(4)r}_{(is)}$ and $fp^{(4)r}_{(is)}$ are, respectively, quantity and price shift variables in foreign demand curves for regional exports.

Governments consume mainly public goods provided by the public administration sectors. Equations (A9) and (A10) show the movement of government consumption in relation to movements in real tax revenue for regional governments and the central government, respectively.

$$x^{(5)r}_{(is)} = taxrev^{r} + f^{(5)r}_{(is)} + f^{(5)r} + f^{(5)}$$
$$i \in G;\ s = 1\text{b}, 2;\ r, b \in S^{*} \qquad \text{(A9)}$$

$$x^{(6)r}_{(is)} = nattaxrev + f^{(6)r}_{(is)} + f^{(6)r} + f^{(6)}$$
$$i \in G;\ s = 1\text{b}, 2;\ r, b \in S^{*} \qquad \text{(A10)}$$

where $x^{(5)r}_{(is)}$ and $x^{(6)r}_{(is)}$ are regional *(5)* and central *(6)* governments demand in region *r* for good *i* from region *s*; $f^{(5)r}_{(is)}$, $f^{(5)r}$ and $f^{(5)}$ are, respectively, commodity and source-specific shift terms for regional governments expenditures in region *r*, shift term for regional governments expenditures in region *r*, and an overall shift term for regional governments expenditures. Similar shift terms ($f^{(6)r}_{(is)}$, $f^{(6)r}$ and $f^{(6)}$) appear in Equation (A10) related to central government expenditures. Finally, $taxrev^{r}$ is the percentage change in real revenue from indirect taxes in region *r*, and $nattaxrev$ refers to the percentage change in aggregate real revenue from indirect taxes, so that government demand moves with endogenous changes in regional and national tax bases.

Equation (A11) specifies the sales tax rates for different users. They allow for variations in tax rates across commodities and their sources and destinations. Tax changes are expressed as percentage-point changes in the *ad valorem* tax rates.

$$t^{(u)r}_{(is)} = f_{i} + f^{(u)}_{i} + f^{(u)r}_{i}$$
$$i \in G;\ s = 1\text{b}, 2;\ b, r \in S^{*};\ u \in U \qquad \text{(A11)}$$

where $t^{(u)r}_{(is)}$ is the power of the tax on sales of the commodity (*is*) to the user (*u*) in region *r*; and f_{i}, $f^{(u)}_{i}$, and $f^{(u)r}_{i}$ are different shift terms allowing percentage changes in the power of tax.

Equations (A12) and (A13) impose the equilibrium conditions in the market's domestic and imported commodities. Notice that there is no margin commodity in the model. Moreover, there is no secondary production in the model. In Equation (A12), demand equals supply for regional domestic commodities.

$$\sum_{j\in H} Y(l,j,r)x_{(l1)}^{(0j)r} = \sum_{(u)\in U} B(l,1b,(u),r)x_{(l1)}^{(u)r}$$
$$l \in G, b, r \in S^* \qquad \text{(A12)}$$

where $x_{(l1)}^{(0j)r}$ is the output of domestic good *l* by industry *j* in region *r*; $x_{(l1)}^{(u)r}$ is the demand of the domestic good *l* by the user *(u)* in region *r*; $Y(l,j,r)$ is the input-output flow measuring the basic value of the output of domestic good *l* by industry *j* in region *r*; and $B(l,1,(u),r)$ is the input-output flow measuring the basic value of domestic good *l* used by *(u)* in region *r*.

Equation (13) imposes zero pure profits in importing. Where $p_{(i(2))}^{(0)}$ is the basic price in the domestic currency of good *i* from foreign source; $p_{(i(2))}^{(w)}$ is the world (Cost, Insurance and Freight) C.I.F. price of imported commodity *i*; phi is the nominal exchange rate; and $t_{(i(2))}^{(0)}$ is the power of the tariff. i.e., one plus the tariff rate, on imports of *i*. Equation (A13) thus, defines the basic price of a unit of imported commodity *i* – the revenue earned per unit by the importer – as the international C.I.F. price converted to domestic currency, including import tariffs.

$$p_{(i(2))}^{(0)} = p_{(i(2))}^{(w)} - phi + t_{(i(2))}^{(0)}$$
$$i \in G \qquad \text{(A13)}$$

Together with Equation (A13), Equations (A14) and (A15) constitute the model's pricing system. The price received for any activity is equal to the costs per unit of output. As can be noticed, the assumption of constant returns to scale adopted here precludes any activity variable from influencing basic prices, i.e., unit costs are independent of the scale at which activities are conducted. Thus, Equation (A14) defines the percentage change in the price received by producers in regional industry *j* per unit of output as equal to the percentage change in *j*'s costs, which are affected by changes in technology and changes in input prices.

$$\sum_{l\in G} Y(l,j,r)\left(p_{(l1)}^{(0)r} + a_{(l1)}^{(0)r}\right) = \sum_{l\in G^*,F}\sum_{s\in S} V(l,s,(1j),r)p_{(ls)}^{(1j)r}$$
$$j \in H;\ r \in S^* \qquad \text{(A14)}$$

where $p_{(l1)}^{(0)r}$ is the basic price of domestic good *i* in region *r*; $a_{(l1)}^{(0)r}$ refer to technological changes, measured as a weighted average of the different types of technical changes with influence on *j*'s unit costs; $p_{(ls)}^{(1j)r}$ is the unit cost of sector *j* in region *r*; $Y(l,j,r)$ is the input-output flow measuring the basic value of the output of domestic good *l* by industry *j* in region *r*; and $V(l,s,(1j),r)$ are input-output flows measuring purchasers' value of good or factor *l* from source *s* used by sector *j* in region *r*.

Equation (A15) imposes zero pure profits in the distribution of commodities to different users. Prices paid for commodity *i* from region *s* in industry *j* in region *r* by each user equate to the sum of its basic value and the costs of the relevant taxes.

$$V(i,s,(u),r)p_{(is)}^{(u)r} = \left(B(i,s,(u),r) + T(i,s,(u),r)\right)\left(p_{(is)}^{(0)} + t_{(is)}^{(u)r}\right)$$
$$i \in G;\ s = 1\text{b}, 2;\ b, r \in S^*;\ u \in U \qquad \text{(A15)}$$

where $p_{(is)}^{(u)r}$ is the price paid by user *(u)* in region *r* for good *(is)*; $p_{(is)}^{(0)}$ is the basic price of domestic good *(is)*; $t_{(is)}^{(u)r}$ is the power of the tax on sales of commodity *(is)* to the user *(u)* in region *r*; $V(i,s,(u),r)$ are input-output flows measuring purchasers' value of good *i* from source *s* used by user *(u)* in region *r*; $B(i,s,(u),r)$ is the input-output flow measuring the basic value of the good *(is)* used by *(u)* in region *r*; and $T(i,s,(u),r)$ is the input-output flow associated with tax revenue of the sales of *(is)* to *(u)* in region *r*.

The theory of investment allocation across industries is represented in Equations (A16) to (A19). The comparative-static nature of the model restricts its use to short-run and long-run policy analysis. When running the model in the comparative-static mode, there is no fixed relationship between capital and investment. Instead, the user decides the required relationship based on the requirements of the specific simulation. Equation (A16) defines the percentage change in the current rate of return on fixed capital in regional sectors. Under static expectations, rates of return are defined as the ratio between the rental values and the cost of a unit of capital in each industry – defined in Equation (A17) –, minus the depreciation rate.

$$r_{(j)}^{r} = \psi_{(j)}^{r}\left(p_{(g+1,2)}^{(1j)r} - p_{(k)}^{(1j)r}\right)$$
$$j \in H;\ r \in S^{*} \qquad \text{(A16)}$$

where $r_{(j)}^{r}$ is the regional-industry-specific rate of return; $p_{(g+1,2)}^{(1j)r}$ is the rental value of capital in sector *j* in region *r*; $p_{(k)}^{(1j)r}$ is the cost of constructing units of capital for regional industries; and $\psi_{(j)}^{r}$ is a regional-industry-specific parameter referring to the ratio of the gross to the net rate of return.

Equation (A16) defines $p_{(k)}^{(1j)r}$ as:

$$V(\bullet,\bullet,(2\mathrm{j}),\mathrm{r})\left(p_{(k)}^{(1j)r} - a_{(k)}^{(1j)r}\right) = \sum_{i\in G}\sum_{s\in S} V(\mathrm{i},\mathrm{s},(2\mathrm{j}),\mathrm{r})\left(p_{(is)}^{(2j)r} - a_{(is)}^{(2j)r}\right)$$
$$j \in H;\ r \in S^{*} \qquad \text{(A17)}$$

where $p_{(is)}^{(2j)r}$ is the price paid by user *(2j)* in region *r* for good *(is)*; $a_{(k)}^{(1j)r}$ and $a_{(is)}^{(2j)r}$ are technical terms; and $V(\mathrm{i},\mathrm{s},(2\mathrm{j}),\mathrm{r})$ represents input-output flows measuring purchasers' value of good *i* from source *s* used by user *(2j)* in region *r*.

Equation (A18) says that if the percentage change in the rate of return in a regional industry grows faster than the national average, capital stocks in that industry will increase at a higher rate than the average national stock. For industries with a lower-than-average increase in their rates of return to fixed capital, capital stocks increase at a lower-than-average rate, i.e., capital is attracted to higher return industries. The shift variable, $f_{(k)}^{(1j)r}$Exogenous in long-run simulation allows shifts in the industry's rates of return.

$$r_{(j)}^{r} - \omega = \varepsilon_{(j)}^{r}\left(x_{(g+1,2)}^{(1j)r} - x_{(g+1,2)}^{(\bullet)r}\right) + f_{(k)}^{(1j)r}$$
$$j \in H;\ r \in S^{*} \qquad \text{(A18)}$$

where $r_{(j)}^{r}$ is the regional-industry-specific rate of return; ω is the overall rate of return on capital; $x_{(g+1,2)}^{(1j)r}$ is the capital stock in industry *j* in region *r*; $f_{(k)}^{(1j)r}$ the capital shift term in sector *j* in region *r*; and $\varepsilon_{(j)}^{r}$ measures the sensitivity of capital growth to rates of return of industry *j* in region *r*.

Equation (A19) implies that the percentage change in an industry's capital stock, $x_{(g+1,2)}^{(1j)r}$, is equal to the percentage change in industry's investments in the period, $z^{(2j)r}$.

$$z^{(2j)r} = x_{(g+1,2)}^{(1j)r} + f_{(k)}^{(2j)r}$$
$$j \in H;\ r \in S^{*} \qquad \text{(A19)}$$

where $f_{(k)}^{(2j)r}$ allows for exogenous shifts in sectorial investments in region *r*.

Equation (A20) defines the regional aggregation of labor prices (wages) across industries in the specification of the labor market. Equation (A21) shows movements in regional wage differentials, $wage_diff^{(r)}$Defined as the difference between the movement in the aggregate regional real wage received by workers and the national real wage.

$$V(\mathrm{g}+1{,}1,\bullet,\mathrm{r})\left(p_{(g+1,1)}^{(\bullet)r}-a_{(g+1,1)}^{(\bullet)r}\right)=\sum\nolimits_{j\in H}V(\mathrm{g}+1{,}1,(1\mathrm{j}),\mathrm{r})\left(p_{(g+1,1)}^{(1j)r}-a_{(g+1,1)}^{(1j)r}\right)$$
$$r\in S^{*} \qquad \text{(A20)}$$

where $p_{(g+1,1)}^{(1j)r}$is the wage in sector j in region r, $a_{(g+1,1)}^{(1j)r}$ is a technical term, and $V(\mathrm{g}+1{,}1,(1\mathrm{j}),\mathrm{r})$ represents input-output flows measuring sectoral labor payments in region *r*.

$$wage\_diff^{(r)}=p_{(g+1,1)}^{(\bullet)r}-cpi-natrealwage$$
$$r\in S^{*} \qquad \text{(A21)}$$

where cpi is the national consumer price index, computed as the weighted average of $p_{(is)}^{(3)r}$ across regions, *r* and consumption goods *(is)*; and $natrealwage$ is the national consumer real wage.

The regional population is defined through the interaction of demographic variables, including interregional migration. Links between regional population and regional labor supply are provided. Demographic variables are usually defined exogenously, and together with the specification of some of the labor market settings, labor supply can be determined together with either interregional wage differentials or regional unemployment rates. In summary, labor supply and wage differentials determine unemployment rates, or labor supply and unemployment rates determine wage differentials.

Equation (A22) defines the percentage-point change in regional unemployment rates in terms of percentage changes in labor supply and employed workers.

$$LABSUP(r)del\_unr^{(r)}=EMPLOY(r)\left(labsup^{(r)}-x_{(g+1,1)}^{(\bullet)r}\right)$$
$$r\in S^{*} \qquad \text{(A22)}$$

where $del\_unr^{(r)}$ measures percentage-point changes in the regional unemployment rate; $labsup^{(r)}$ is the variable for regional labor supply; and the coefficients $LABSUP(r)$ and $EMPLOY(r)$ are the benchmark values for regional labor supply and regional employment, respectively. The variable $labsup^{(r)}$ moves with regional workforce participation rate, proportional to the regional population and population of working age. Equation (A23) defines regional population changes in the model as ordinary changes in flows of net regional migration ($d\_rm^{(r)}$), net foreign migration ($d\_fm^{(r)}$), and natural population growth ($d\_g^{(r)}$).

$$POP(r)pop^{(r)}=d\_rm^{(r)}+d\_fm^{(r)}+d\_g^{(r)}$$
$$r\in S^{*} \qquad \text{(A23)}$$

where $POP(r)$ is a coefficient measuring regional population in the benchmark year.

Equation (A24) shows movements in per-household utility differentials, $util\_diff^{(r)}$, defined as the difference between the movement in regional utility and the overall national utility (agg_util), including a shift variable, $futil^{(r)}$.

$$util\_diff^{(r)} = utility^{(r)} - agg\_util + futil^{(r)}$$
$$r \in S^* \quad \text{(A24)}$$

Finally, we can define changes in regional output as weighted averages of changes in regional aggregates, according to Equation (A25) below:

$$GRP^r grp^r = C^r x_{(\bullet\bullet)}^{(3)r} + INV^r z^{(2\bullet)r} + GOV^{(5)r} x_{(\bullet\bullet)}^{(5)r} + GOV^{(6)r} x_{(\bullet\bullet)}^{(6)r} + \left(FEXP^r x_{(\bullet\bullet)}^{(4)r} - FIMP^r x_{(\bullet 2)}^{(\bullet)r}\right) + \left(DEXP^r x_{(\bullet(1r))}^{(\bullet)s} - DIMP^r x_{(\bullet(1s))}^{(\bullet)r}\right)$$
$$r \in S^*; s \in S^* \text{ for } s \neq r \quad \text{(A25)}$$

where grp^r is the percentage change in real Gross Regional Product in region *r*; and the coefficients GRP^r, INV^r, $GOV^{(5)r}$, $GOV^{(6)r}$, $FEXP^r$, $FIMP^r$, $DEXP^r$, and $DIMP^r$ respectively, the following regional aggregates: investments, regional governments spending, central government spending, foreign exports, foreign imports, domestic exports, and domestic imports. National output, GDP, is, thus, the sum of GRP^r across all regions *r*. Notice that regional domestic trade balances cancel out.

To close the model, we set the following variables exogenously, which are usually exogenous both in short-run and long-run simulations: $a_{(g+1,s)}^{(1j)r}$, $a_{(i)}^{(1j)r}$, $a_{(i)}^{(2j)r}$, $a_{(i\bullet)}^{(3)r}$, $fq_{(is)}^{(4)r}$, $fp_{(is)}^{(4)r}$, $f_{(is)}^{(5)r}$, $f^{(5)r}$, $f^{(5)}$, $f_{(is)}^{(6)r}$, $f^{(6)r}$, $f^{(6)}$, f_i, $f_i^{(u)}$, $f_i^{(u)r}$, $p_{(i(2))}^{(w)}$, $t_{(i(2))}^{(0)}$, $a_{(l1)}^{(0)r}$, $a_{(k)}^{(1j)r}$, $a_{(is)}^{(2j)r}$, $a_{(g+1,1)}^{(\bullet)r}$, ω, $f_{(k)}^{(2j)r}$, $d\_fm^{(r)}$, $d\_g^{(r)}$, and $futil^{(r)}$. To complete the short run environment, **we also set unchanged current stocks of capital ($x_{(g+1,2)}^{(1j)r}$)**, the national real wage ($natrealwage$), regional wage differentials, ($wage\_diff^{(r)}$), and regional population, by keeping regional migration unchanged ($d\_rm^{(r)}$).[3]

There are other definitions of variables computed by using outcomes from simulations based on equations (A1)-(A25). Of particular interest to our discussion is the definition of regional/national GDP and its components.

Calibration

The calibration of the model requires two subsets of data to define its numerical structure to implement the model empirically. First, we need information from an absorption matrix derived from interregional input-output sources (Supplementary Table 2) to calculate the coefficients of the model based on the following input-output flows:

- $B(i, 1b, (u), r)$, with $i \in G^*, (u) \in U, b, r \in S^*$
- $M(i, s, (u), r)$, with $i \in G^*, s \in S, (u) \in U, r \in S^*$[4]
- $T(i, s, (u), r)$, with $i \in G^*, s \in S, (u) \in U, r \in S^*$
- $V(i, s, (u), r)$, with $i \in G^*, s \in S, F, (u) \in U, r \in S^*$
- $Y(i, j, r)$, with $i \in G^*, j \in H, r \in S^*$

[3] In a long run closure, the assumptions on interregional mobility of capital and labor are relaxed by swapping variables $x_{(g+1,2)}^{(1j)r}$, $natrealwage$, $wage\_diff^{(r)}$ and $d\_rm^{(r)}$, for $f_{(k)}^{(1j)r}$, $del\_unr^{(r)}$ and $util\_diff^{(r)}$.

[4] In the presentation of the model, we have included margin-commodities, $M(i, s, (u), r)$, in $B(i, 1b, (u), r)$.

We complete this information with supplementary demographic data from the *Instituto Nacional de Estadísticas* (INE) to calibrate the coefficients $LABSUP(r)$, $EMPLOY(r)$ and $POP(r)$, with $r \in S^*$. Because these estimates are based on snapshot observations for a single year revealing the economic structure of the economic system, this subset of data is denoted "structural coefficients" (Haddad et al., 2002).

The second piece of information necessary to calibrate the model is represented by the subset of data defining various parameters, mainly elasticities. These are called "behavioral parameters". Empirical estimates for some of the parameters of the model are not available in the literature. We have thus relied on "best guesstimates" based on typical values employed in similar models. The current version of the model runs under constant returns to scale so that we set to 1.0 the values of $\mu_{(g+1,\bullet)}^{(1j)r}$ in Equation (A4). The marginal budget shares in regional household consumption, $\beta_{(i)}^{r}$ in Equation (A6), were calibrated from the input-output data, assuming the average budget share to be equal to the marginal budget share, and the subsistence parameter $\gamma_{(i)}^{r}$, also in Equation (A6), it was associated with a Frisch parameter equal to -1.0824. The ratio of gross to the net rate of return, $\psi_{(j)}^{r}$ in Equation (A16), was set to 1.20. Finally, we set to 3.02 the parameter for the sensitivity of capital growth to rates of return, $\varepsilon_{(j)}^{r}$ in Equation (A18). Other behavioral parameters taken from the literature are presented in Supplementary Table 3.

Supplementary Table 2 Aggregate Flows in the Absorption Matrix: Chile, 2014 (values in current CHP billions)

LABELS	User (1j)^r	User (2j)^r	User (3)^r	User (4)	User (5)^r	User (6)^r	User (7)	TOTAL
i G, s S*	B(i,1b,(1j),r)	B(i,1b,(2j),r)	B(i,1b,(3),r)	B(i,1b,(4))	B(i,1b,(5),r)	B(i,1b,(6),r)	B(i,1b,(7))	B(i,1b,(•),•)
i G, s S-S*	B(i,2,(1j),r)	B(i,2,(2j),r)	B(i,2,(3),r)	B(i,2,(4))	B(i,2,(5),r)	B(i,2,(6),r)	B(i,2,(7))	B(i,2,(•),•)
i G, s S	T(i,s,(1j),r)	T(i,s,(2j),r)	T(i,s,(3),r)	T(i,s,(4))	T(i,s,(5),r)	T(i,s,(6),r)	-	T(i,s,(•),•)
s F	V(g+1,s,(1j),r)	-	-	-	-	-	-	V(g+1,s,(•),•)
TOTAL	Y(•,•,r)	V(•,•,(2j),r)	V(•,•,(3),r)	V(•,•,(4))	V(•,•,(5),r)	V(•,•,(6),r)	V(•,•,(7))	V(•,•,(•),•)

2014	User (1j)^r	User (2j)^r	User (3)^r	User (4)	User (5)^r	User (6)^r	User (7)	TOTAL
i G, s S*	103,251	28,961	68,876	47,982	2,803	16,050	-28	267,897
i G, s S-S*	27,263	5,376	14,590	0	19	113	0	47,360
i G, s S	3,109	1,108	10,269	2,294	14	81	-	16,875
s F	134,273	-	-	-	-	-	-	134,273
TOTAL	267,897	35,445	93,735	50,277	2,836	16,244	-28	466,406

Source: Haddad, E. A. et al. (2018)

Supplementary Table 3 Behavioral parameters used in the BMCH model

Sector	Regional trade elasticities (Equation A1)	International trade elasticities (Equation A2)	Elasticity of substitution between primary factors (Equation A3)	Export demand elasticities (Equation A8)
S1	1.866	1.866	1.20	-3.560
S2	0.955	0.955	0.80	-0.160
S3	2.939	2.939	0.80	-2.213
S4	2.800	2.800	0.60	0.000
S5	1.900	1.900	0.60	0.000
S6	1.900	1.900	0.60	-1.770
S7	1.900	1.900	0.60	-1.770
S8	1.900	1.900	0.60	-1.770
S9	1.900	1.900	0.60	-1.770
S10	1.900	1.900	0.60	-1.770
S11	1.900	1.900	0.60	-1.770
S12	1.900	1.900	0.60	0.000

Supplementary References

1. Cardona, O. D., Ordaz, M., Reinoso, E. & Yamin, L. *CAPRA – Comprehensive Approach to Probabilistic Risk Assessment: International Initiative for Risk Management Effectiveness*. 15th World Conference of Earthquake Engineering, Lisboa, Portugal (2012).
2. ERN-AL. CAPRA, *Methodology of Probabilistic Risk Assessment*. Obtained from: http://www.ecapra.org/. Accessed 1 April 2020.
3. Esteva, L. *Criteria for the Construction of Spectra for Seismic Design*. 3rd Pan-American Symposium of Structure (in Spanish), (pages. 3-8). Caracas, Venezuela (1967).
4. Esteva, L. *Seismic Regionalization of Mexico for Engineering Purposes*. Instituto de Ingeniería. Universidad Nacional Autónoma de México (1970).
5. Cornell, C. A. Engineering Seismic Risk Analysis. *Bulletin Seismological Society of America* **58**(5):1583–1606 (1968).
6. Centro Regional de Sismología para América del Sur (CERESIS). *Catálogo sísmico regional de parámetros focales para América del Sur y mapa con el resultado del cálculo de peligro sísmico* (1997).
7. Youngs R., Chiou S., Silva W. & Humprey J. Strong ground motions attenuation relationships for subduction zone earthquakes. *Seismological Research Letters* **64**, 58–73, 1997.
8. Sadigh, K., Chang, C.-Y., Egan, J. A., Makdisi,F. & Youngs, R. R.; Attenuation Relationships for Shallow Crustal Earthquakes Based on California Strong Motion Data. *Seismological Research Letters* **68**(1), 180–189 (1997).
9. Gunasekera, R., Ishizawa, O., Aubrecht, C., Blankespoor, B., Murray, S., Pomonis, A. & Daniel, J. Developing an Adaptive Global Exposure Model to Support the Generation of Country Disaster Risk Profile. *Earth-Science Reviews* **150**, 594–608 (2015).
10. Aubrecht C. & León Torres, J. A. *Top-Down Identification of Mixed vs. Residential Urban Areas: Evaluation of Remotely Sensed Nighttime Lights for a Case Study in Cuenca City, Ecuador*. In Proceeding of the 1st Int. Electron. Remote Sens., Sciforum Electronic Conference Series, Vol. 1 (2015).
11. Aubrecht, C. & León Torres, J. A. Evaluating Multi-sensor Nighttime Earth Observation Data for Identification of Mixed vs. Residential Use in Urban Areas. *Remote Sens* **8**(114), 1–19 (2016).
12. Pomonis, A. *Estimation of Residential Inventory and Exposure in Urban and Rural Areas for Disaster Loss Estimation*. The World Bank - Social, Urban, Rural & Resilience (GSURR), Disaster Risk Management (DRM): Washington DC, USA (2014).
13. León Torres, J. A. *City Disaster Risk Profile: Tegucigalpa City*. Washington DC, USA: The World Bank (2019).
14. Aubrecht, C., Steinnocher, K., Köstl, M., Züger. J. & Loibl, W. Long-term spatiotemporal social vulnerability variation considering health-related climate change parameters particularly affecting the elderly. *Nat. Hazards* **68**(3), 1371–1384 (2013).
15. Aubrecht, C., Gunasekera, R. & Ishizawa, O. *Urban characterization aspects, and associated spatial scale considerations in the context of global-level exposure modeling: Comparative data evaluation*. In Proceedings of the Second European Conference on Earthquake Engineering and Seismology (2ECEES), Istanbul, Turkey, 24–29 August 2014.
16. Reinoso E. & Jaimes M. Estimation of Earthquake Life Loss Computed from the Expected Probabilistic Building Loss. *Journal of Earthquake Engineering*, UEQE-2012-1519 (2012).
17. Alvarez, C., Rivera, F., Hube, M., Santa María, H. & Acosta, A.. *Chile Exposure and Vulnerability*. South America Risk Assessment Project. GEM (2015).
18. Ordaz, M., Miranda, E., Reinoso, E. & Pérez-Rocha, L. *Seismic Loss Estimation Model for Mexico City*. 12th World Conference on Earthquake Engineering, Auckland, New Zealand (2000).
19. ERN-AL. CAPRA, Vulnerability of Buildings and Infrastructure (2010). http://www. ecapra.org/. Accessed 10 April 2020.
20. León, J. A. *A Probabilistic Model of the Propagation of Earthquake Losses through the Economy*. Ph.D. Thesis. Universidad Nacional Autónoma de México (2021).
21. Haddad, E. A. & Hewings, G. J. Market Imperfections in a Spatial Economy: some Experimental Results. *Quarterly Review of Economics and Finance* **45**(2–3):476–496 (2005).
22. Peter, M. W., Horridge, M., Meagher, G. A., Naqvi, F. & Parmenter, B. R. *The Theoretical Structure of Monash-MRF*. Monash University, Clayton: Working paper No. OP-85, IMPACT Project (1996).
23. Haddad, E., Domingues, E. & Perobelli, F. Regional effects of economic integration: the case of Brazil. *Journal of Policy Modeling* **24**(5), 453-482 (2002).
24. Haddad, E., Aroca, P., Arantes, S., Días, L., Fernandes, R., Li, D., Pimenta, B., Rocha, A., Sass, K. & Ussumi, K. *Interregional Input-Output System for Chile*. The University of Sao Paulo, Regional and Urban Economics Lab (NEREUS) (2018).
