# Supplementary material for: Risk caused by the propagation of earthquake losses through the economy
Source: Nat Commun. 2022 May 25;13:2908. doi: 10.1038/s41467-022-30504-3 (PMC9132971; doi:10.1038/s41467-022-30504-3)
Supplement: Supplementary file 2 — Reporting summary [file 41467_2022_30504_MOESM2_ESM.pdf]

nature portfolio

Corresponding author(s): Dr. José A. León

Last updated by author(s): Mar 18, 2022

Reporting Summary

Nature Portfolio wishes to improve the reproducibility of the work that we publish. This form provides structure for consistency and transparency in reporting. For further information on Nature Portfolio policies, see our Editorial Policies and the Editorial Policy Checklist.

Statistics

For all statistical analyses, confirm that the following items are present in the figure legend, table legend, main text, or Methods section.

n/a	Confirmed	
☒	☐	The exact sample size (*n*) for each experimental group/condition, given as a discrete number and unit of measurement
☒	☐	A statement on whether measurements were taken from distinct samples or whether the same sample was measured repeatedly
☒	☐	The statistical test(s) used AND whether they are one- or two-sided<br>*Only common tests should be described solely by name; describe more complex techniques in the Methods section.*
☒	☐	A description of all covariates tested
☒	☐	A description of any assumptions or corrections, such as tests of normality and adjustment for multiple comparisons
☐	☒	A full description of the statistical parameters including central tendency (e.g. means) or other basic estimates (e.g. regression coefficient) AND variation (e.g. standard deviation) or associated estimates of uncertainty (e.g. confidence intervals)
☒	☐	For null hypothesis testing, the test statistic (e.g. *F*, *t*, *r*) with confidence intervals, effect sizes, degrees of freedom and *P* value noted<br>*Give P values as exact values whenever suitable.*
☒	☐	For Bayesian analysis, information on the choice of priors and Markov chain Monte Carlo settings
☒	☐	For hierarchical and complex designs, identification of the appropriate level for tests and full reporting of outcomes
☒	☐	Estimates of effect sizes (e.g. Cohen's *d*, Pearson's *r*), indicating how they were calculated

Our web collection on statistics for biologists contains articles on many of the points above.

Software and code

Policy information about availability of computer code

Data collection	no sotware was used.
Data analysis	Data analysis was performed my means of the program DIRAS-2020, software specifically created to carry out probabilistic risk calculations based on the information coming from a conventional seismic risk model and a spatial CGE model. DIRAS interacts with the program CRunGEM, which is an environment for running CGE models built with the software GEMPACK. DIRAS can be freely downloaded from the public repository (https://github.com/JALeonTorres/RAPELE-). This repository includes an hands-on guideline to reproduce all results presented in this study. CRunGEM - version 2015 can be downloaded from (https://www.copsmodels.com/crungem.htm) and GEMPACK version 12.1 from (https://www.copsmodels.com/gpeidl.htm). For reproducing the results, it is just required a temporary license available with the trial version of GEMPACK v12.1.

For manuscripts utilizing custom algorithms or software that are central to the research but not yet described in published literature, software must be made available to editors and reviewers. We strongly encourage code deposition in a community repository (e.g. GitHub). See the Nature Portfolio guidelines for submitting code & software for further information.

Data

Policy information about availability of data

All manuscripts must include a data availability statement. This statement should provide the following information, where applicable:

- Accession codes, unique identifiers, or web links for publicly available datasets
- A description of any restrictions on data availability
- For clinical datasets or third party data, please ensure that the statement adheres to our policy

The data that support the findings of this study include the seismic hazard model of Chile, vulnerability functions, the exposure model of non-residential buildings of

Chile, and of the Interregional General Equilibrium Model for Chile (BMCH) all freely available from (https://github.com/JALeonTorres/RAPELE-). For the case of the non-residential buildings model of Chile, data include the Land Scan dataset (2017 version), available at (https://landscan.ornl.gov/), the GHS-POP dataset (2015 version), available at (https://ghsl.jrc.ec.europa.eu/), the WorldPop dataset (version 2015), available at (https://www.worldpop.org/) and the nighttime scenes from VIIRS sensor (version 1, updated March 2017), available at (https://earthdata.nasa.gov/earth-observation-data/near-real-time/download-nrt-data/viirs-nrt).

Field-specific reporting

Please select the one below that is the best fit for your research. If you are not sure, read the appropriate sections before making your selection.

☐ Life sciences ☐ Behavioural & social sciences ☒ Ecological, evolutionary & environmental sciences

For a reference copy of the document with all sections, see nature.com/documents/nr-reporting-summary-flat.pdf

Ecological, evolutionary & environmental sciences study design

All studies must disclose on these points even when the disclosure is negative.

Study description	The study presents for the first time a set of probabilistic risk indicators regarding losses of production, employment, GDP, gross regional product, export volume, inflation, tariff revenue, among others, due to earthquakes. All indicators are computed both at country and regional level by using a systematic probabilistic approach, which considers a vast collection of stochastic events that collectively describe the entire seismic hazard of a country. The novel approach is applied to Chile, country frequently affected by large earthquakes and presenting remarkable regional economic disparities.
Research sample	The research sample includes datasets of the seismic hazard model of Chile, vulnerability functions, the exposure model of non-residential buildings of Chile, and of the Interregional General Equilibrium Model for Chile (BMCH), all freely available at https://github.com/JALeonTorres/RAPELE-. For the case of the non-residential buildings model of Chile, the research sample includes the Land Scan dataset (https://landscan.ornl.gov/) , the GHS-POP dataset (https://ghsl.jrc.ec.europa.eu/), the WorldPop dataset (https://www.worldpop.org/) and the nighttime scenes dataset from VIIRS sensor (https://earthdata.nasa.gov/earth-observation-data/near-real-time/download-nrt-data/viirs-nrt).
Sampling strategy	The sample included the essential and necessary components to carry out a seismic risk model of Chile which are a seismic hazard model, an exposure model of assets and a component of seismic vulnerability. Furthermore, to analyze the interactions among different economic sectors of the Country, the sample included an interregional computable general equilibrium model for Chile.
Data collection	Data were collected by the authors from the websites reported in previous sections.
Timing and spatial scale	The seismic hazard model of Chile has a 30 kilometers spatial resolution and used a seismic catalog with events from 1900 to 2018. The seismic vulnerability component employed vulnerability functions developed in 2019 at the Insitituto de Ingenieria of UNAM. The exposure model of non-residential buildings of Chile used data collected in 2019 from LandScan, WorldPop, GHS and VIIRS (spatial resolution approximately 1 Km). The interregional general equilibrium model for Chile used economic data from 2014 and the model spatially distinguishes each one of the 15 regions of Chile. The data used in this study correspond to the most current versions that were available and accessible at the time of the investigation.
Data exclusions	No data were excluded from the analysis.
Reproducibility	To guarantee the reproducibility of the results, it is provided all the necessary input data, piece of software and a guideline with precise instructions to reproduce the results.
Randomization	It is not relevant for this study. This study presents a new set of risk indicators which are applicable to any country affected by earthquakes.
Blinding	Blinding is no relevant. This study presents a new set of risk indicators which are applicable to any country affected by earthquakes.

Did the study involve field work? ☐ Yes ☒ No

Reporting for specific materials, systems and methods

We require information from authors about some types of materials, experimental systems and methods used in many studies. Here, indicate whether each material, system or method listed is relevant to your study. If you are not sure if a list item applies to your research, read the appropriate section before selecting a response.

Materials & experimental systems

n/a	Involved in the study
☒	☐ Antibodies
☒	☐ Eukaryotic cell lines
☒	☐ Palaeontology and archaeology
☒	☐ Animals and other organisms
☒	☐ Human research participants
☒	☐ Clinical data
☒	☐ Dual use research of concern

Methods

n/a	Involved in the study
☒	☐ ChIP-seq
☒	☐ Flow cytometry
☒	☐ MRI-based neuroimaging
